# Dynamic of bifurcation, chaotic structure and multi soliton of fractional nonlinear Schrödinger equation arise in plasma physics

**DOI:** 10.1038/s41598-024-72744-x

**Published:** 2024-10-28

**Authors:** Ibtehal Alazman, Badr Saad Thaar Alkahtani, Manvendra Narayan Mishra

**Affiliations:** 1 Department of Mathematics and Statistics, College of Science, Imam Mohammad Ibn Saud Islamic University (IMSIU), 13318 Riyadh, Saudi Arabia; 2 Department of Mathematics, College of Science, King Saud University, 11989 Riyadh, Saudi Arabia; 3 Department of Mathematics, Suresh Gyan Vihar University, Jaipur, Rajasthan India

**Keywords:** Soliton solutions, FNLSE, Truncated M-fractional derivative, NEDAM, Stability property, Sensitivity analysis, Bifurcation and chaos, Applied mathematics, Mathematics and computing

## Abstract

In this study, we examine the third-order fractional nonlinear Schrödinger equation (FNLSE) in $(1+1)$-dimensional, by employing the analytical methodology of the new extended direct algebraic method (NEDAM) alongside optical soliton solutions. In order to better understand high-order nonlinear wave behaviors in such systems, the researched model captures the physical and mathematical properties of nonlinear dispersive waves, with applications in plasma physics and optics. With the aid of above mentioned approach, we rigorously assess the novel optical soliton solutions in the form of dark, bright–dark, dark–bright, periodic, singular, rational, mixed trigonometric and hyperbolic forms. Additionally, stability assessments using conserved quantities, such as Hamiltonian property, and consistency checks were used to validate the solutions. The dynamic structure of the governing model is further examined using chaos, bifurcation, and sensitivity analysis. With the appropriate parameter values, 2D, 3D, and contour plots can all be utilized to graphically show the data. This work advances our knowledge of nonlinear wave propagation in Bose–Einstein condensates, ultrafast fibre optics, and plasma physics, among other areas with higher-order chromatic effects.

## Introduction

The ability of Nonlinear Partial Differential Equations (NLPDEs) to capture nonlinear interactions and dynamic behaviours makes them indispensable tools for both theoretical research and practical applications^1^. On the contrary, NLPEs proposed solutions that are opposite to their linear complements, these NLPDEs resist direct superposition principles that result in escalating an elegant array of dynamics that includes complicated spatial-temporal patterns, chaos, and bifurcations^2^. These models result in demonstrating various physical phenomena including optics, heat transfer, and fluid dynamics, in which collaboration of nonlinear parts leads to a level of complication that reveals the non-linearity of the basic physical advancements. Solitary wave solutions, also known as solitons, are fascinating and ubiquitous phenomena that arise in different branches of physics, such as nonlinear optics, plasma physics, solid-state physics, and fluid dynamics^3^. Solitons are used in long-distance optical fiber communications for their ability to propagate without distortion, enabling high-speed data transmission over vast distances^4^. Optical solitons are self-reinforcing wave packets that propagate without significant change in shape due to a balance between nonlinear and dispersive effects. They play a crucial role in various optical systems and have applications in telecommunications, fiber optics, and nonlinear optics. The dynamics of soliton propagation across different kinds of optical waveguides, including photonic crystal fibres, metamaterials, couplers, and fibres, are governed by different models. In this case, the Nonlinear Schrödinger equation (NLSE) is quite important. In many fields of science and engineering, including as quantum mechanics, optics, laser technology, ultracold physics, and biology, the NLSE is crucial^5,6^. Its solutions spur advancements in a variety of scientific and technological domains and offer insightful knowledge about the behaviour of intricate systems controlled by nonlinear wave equations.

In addition, many scientific domains and engineering applications depend on fractional calculus (FC), a branch of mathematical analysis that deals with integrals and derivatives of non-integer order. FC is essential in the study of ultrashort pulse propagation in optical fibers due to its ability to accurately model nonlocal dispersion and nonlinear effects, characterize anomalous dispersion, analyze pulse compression techniques, design fiber optic systems, enhance accuracy and predictability, and advance ultrafast photonics research. Ultrashort pulses often experience anomalous dispersion in optical fibers, leading to pulse broadening and distortion. Fractional calculus allows for the modeling of anomalous dispersion more effectively, leading to better predictions of pulse dynamics. Many natural processes and systems exhibit behaviors that cannot be adequately described using classical integer-order calculus. Its uses span basic science to useful engineering solutions, advancing science, technology, and business.cites8.

The NLSE is a fundamental equation used to describe the propagation of light pulses in optical fibers, especially when dealing with ultrashort pulses^7^. NLSE is extensively used to model the propagation of optical pulses in nonlinear optical fibers. Higher-order dispersion terms in the NLSE account for additional dispersion effects beyond the first-order group velocity dispersion, allowing for more accurate modeling of pulse dynamics, especially for ultrashort pulses. NLSE^8,9^ simulations aid in the design and optimization of mode-locked fiber lasers, which generate ultrashort pulses for various applications, including spectroscopy, materials processing, and medical imaging. For a complete analysis of dynamics and the application of NLPDEs to real-world problems, analytical solutions are crucial. Over time, a variety of effective methods have been created to create analytical solutions for NLPDEs. Many scientists and researchers employ several successful strategies, such as Khater method^10^, the extended simple equation method^11^, the modified exponential function method^12^, Lie symetric analysis^13^, the Hirota bilinear method^14^, Heir equation method^15^, the adomian decomposition method^16^, and improved *F*-expansion method^17^ and the Darboux transformation method^18^ to enhance their comprehension of real-world phenomena. Compared to the most recent published approaches in the literature^19–24^, the NEDAM has a number of important advantages. It performs admirably in producing new and varied solutions, boosts computational effectiveness, exhibits increased application versatility, and produces excellent results that are essential for comprehending intricate nonlinear dynamics. Because of this, it is a better approach to solving a variety of nonlinear differential equations in contemporary research. This method is designed to extend the capabilities of traditional direct algebraic methods by incorporating additional algebraic structures or operators. This innovation can uncover solutions that existing methods might not reveal. While methods like the auxiliary equation method and Jacobi elliptic function method are effective in producing soliton and periodic solutions, they may not always capture the full range of potential solutions, especially in complex systems^25^.

Notwithstanding the existing body of research on the third-order FNLSE, there is a pressing need for comprehensive investigations that amalgamate of analytical methodologies to provide precise solutions^26,27^. The precision and variety of these solutions are critical for tackling a wide range of nonlinear phenomena. This study aims to bridge this knowledge gap by employing the NEDAM to analyze the FNLSE. This paper aims to improve the accuracy and reliability of its solutions by thoroughly analysing the third-order FNLSE and proposing an efficient technique for its resolution. Also, the bifurcation anlysis and sensitive analysis of the proposed model is analysis. These investigations have produced a wide range of soliton solutions that have not been reported in earlier literature.

The third order NLSE, proposed in^28^, is a similar physical model that meets the aforementioned characteristics.

1 $$\begin{aligned} i(D^{\alpha ,\beta }_{M,t}\Theta + D^{3\alpha ,\beta }_{M,x}\Theta )+|\Theta |^{2}(\wp _{1} \Theta +i \wp _{2} D^{\alpha ,\beta }_{M,x}\Theta )+i \wp _{3} (D^{\alpha ,\beta }_{M,x}|\Theta |^{2}) \Theta =0. \end{aligned}$$

The complex function $\Theta (x,t)$ describes how light propagates in optical fibres when ultrashort pulses are generated. Also, $\wp _{1},~\wp _{2}$ and $\wp _{3}$ are non-zero constants. A fundamental tool in plasma physics and optics, the NLSE sheds light on how nonlinear waves behave. Because of its capacity to simulate the intricate relationship between nonlinearity and dispersion, it is essential for expanding theoretical knowledge and creating useful applications in these domains. The NLSE is still a useful tool for scientists and engineers, whether they are studying plasma waves or optical fiber communications. Recently, many acadamia studied the NLSE by applying different anomirious approaches for example, Akram et al.^29^ studied the perturbed NLSE aris in optics by encomposing the improved *F*-expansion method extract different soliton solution. Shehata and Bekir^30^, reported bright and dark from the NLSE. Mathanaranjan et al.^31^ employed the new extended auxiliary equation method of the NLSE with nonlinear chromatic dispersion and extract optical soliton solutions. Ali et al.^32^ used the new extended unified auxiliary equation method of the high order NLSE and find the solitary wave solutions.

This article is organized in the following manner: The features of the chosen derivative are discussed in “The M-truncated derivative” section). The methodology of the NEDAM are discussed in (“Mathodolgy” section). In “Applications of NEDAM” section illustrates the method’s applications. In Stability property section, the stability property is described. The bifurcation and sensitive analysis are illustrated in “Bifurcation analysis” section. The physical interpretations of results are provided in “Simulation and discussion” section. Lastly, in “Conclusion” section synthesizes the conclusion.

## The *M*-truncated derivative

A useful mathematical tool for modelling intricate dynamic systems and examining non-local interdependence in data is the *M* truncated derivative. By truncating the fractional derivative’s series expansion, the *M*-truncated fractional derivative is an effective technique that makes fractional calculus useful in real-world applications. It is especially useful in disciplines where fractional order models are employed to explain complex systems because of its capacity to strike a compromise between computational efficiency and the requirement to capture crucial fractional dynamics. Because of its derivative, fractional calculus may be used practically in real-world applications, which makes it an indispensable tool for both theoretical and applied study. Its application is multidisciplinary, advancing knowledge and forecasting across a range of sectors.

### Definition

For a function ${\mathfrak {f}} :(0,\infty ){\rightarrow }R$, the truncated *M*-fractional derivative^33^ of order $\alpha$ is described as follows:

2 $$\begin{aligned} D^{\alpha ,\beta }_M g[\chi ]=\lim \limits _{\epsilon \rightarrow 0}\frac{g(\chi E_\beta (\epsilon \chi ^{1-\alpha })-g(\chi ))}{\epsilon },~\chi>0,~~ \forall ~0<\alpha \le 1,~~\beta >0, \end{aligned}$$

as well as a Mittag–Leffler function, $E_\beta (.)$.

### Theorem 1

Let $\beta >0$, $\alpha \in (0,1]$, ${\mathfrak {g}}={\mathfrak {g}}(\chi )$, and ${\mathfrak {f}}={\mathfrak {f}}(\chi )$ be assumed, at a location $\chi >0$ be $\alpha$-differentiable, the selected derivative has the following characteristics:

(i) $$D_{M}^{{\alpha ,\beta }} (a{\mathfrak{g}} + b{\mathfrak{h}}) = aD_{M}^{{\alpha ,\beta }} {\mathfrak{g}} + bD_{M}^{{\alpha ,\beta }} {\mathfrak{h}},~\forall a,~b \in R,$$
(ii) $$D_{M}^{{\alpha ,\beta }} (c) = 0,~\;{\text{where}}\;{\text{the}}\;{\text{constant}}\;{\text{is}}\;{\text{c}},$$
(iii) $$D_{M}^{{\alpha ,\beta }} ({\mathfrak{g}}.{\mathfrak{h}}) = {\mathfrak{g}}D_{M}^{{\alpha ,\beta }} ({\mathfrak{h}}) + {\mathfrak{h}}D_{M}^{{\alpha ,\beta }} ({\mathfrak{g}}),$$
(iv) $$D_{M}^{{\alpha ,\beta }} \left( {\frac{{\mathfrak{g}}}{{\mathfrak{h}}}} \right) = \frac{{{\mathfrak{h}}D_{M}^{{\alpha ,\beta }} ({\mathfrak{g}}) - {\mathfrak{g}}D_{M}^{{\alpha ,\beta }} ({\mathfrak{h}})}}{{{\mathfrak{h}}^{2} (\chi )}}.$$

Also, $D^{\alpha ,\beta }_M({\mathfrak {f}}(\chi ))=\frac{\chi ^{1-\alpha }}{\Gamma (\beta +1)}\frac{d{\mathfrak {f}}}{d \chi }$, in the case where the function ${\mathfrak {f}}(\chi )$ is differentiable.

## Mathodolgy

Analytical approaches are crucial tools for extending NLPDE knowledge, analysis, and application across disciplinary boundaries. They provide useful information, encourage creativity, make it easier to create models, enable numerical simulations, and advance both the theoretical and practical aspects of NLPDE research. Let us suppose the nonlinear fractional equation:

3 $$\begin{aligned} {\mathbb {G}}(\Gamma ,~ D^{\alpha ,\beta }_{M,t} \Gamma ,~D^{\alpha ,\beta }_{M,x} \Gamma ,~D^{\alpha ,\beta }_{M,2t} \Gamma ,~D^{\alpha ,\beta }_{M,2x} \Gamma ,~ \ldots )=0. \end{aligned}$$

Firstly, apply the wave transformation to get the nonlinear ordinary differential equations (ODEs):

4 $$\begin{aligned} \Gamma (x,t)= & \Upsilon (\chi )e^{i( \phi )}. \end{aligned}$$

Where, $\chi =\frac{\Gamma (\beta +1) \left( x^{\alpha }-\vartheta t^{\alpha }\right) }{\alpha },~\phi =-\frac{\Gamma (\beta +1) \left( \kappa x^{\alpha }-\delta \vartheta t^{\alpha }\right) }{\alpha }.$ Here $\Upsilon (\chi )$ denotes the amplitude and $\vartheta ,~\kappa ,~\delta$ refers as nonzero constants. We get the following nonlinear ordinary differential equations (ODE):

5 $$\begin{aligned} {\mathbb {Q}}( \Upsilon ,~\Upsilon ',~\Upsilon {''},~\ldots )=0. \end{aligned}$$

Assume the general solutions of Eq. (5) as below:

6 $$\begin{aligned} \Upsilon (\chi ) = \sum _{i=1}^{n}\big [\alpha _{i} {\mathcal {H}}(\chi )^i\big ]. \end{aligned}$$

Where $\alpha _i (i = 0,~ 1,~ 2,\ldots ,n)$ are constants to be addressed later and. Where the function ${\mathcal {H}}(\chi )$ satisfies the Riccati equation as:

7 $$\begin{aligned} ({\mathcal {H}}'(\chi ))= Ln(a) (\gamma _{1}+ \gamma _{2} \mathcal {H(\chi )}+\gamma _{3}({\mathcal {H}}(\chi )^{2}),~a\ne 0,1. \end{aligned}$$

Where $\gamma _{i},~i={1,2,3}$ are real constants and ${\mathbb {D}}=\gamma _2^2-4 \gamma _1 \gamma _3<0$. The Eq. (6) has following solutions:

- (1): If $$\gamma _2^2-4 \gamma _1 \gamma _3<0$$, and $$\gamma _3 \ne 0$$,
  8 $$\begin{aligned} {\mathcal {H}}_{1}=-\frac{\gamma _{2}}{2 \gamma _{3}}+\frac{\sqrt{-{\mathbb {D}}}}{2\gamma _{3}}\tan _{a}\left( \frac{\sqrt{-{\mathbb {D}}}}{2}\chi \right) , \end{aligned}$$
  9 $$\begin{aligned} {\mathcal {H}}_{2}=-\frac{\gamma _{2}}{2 \gamma _{3}}+\frac{\sqrt{-{\mathbb {D}}}}{2\gamma _{3}}\cot _{a}\left( \frac{\sqrt{-{\mathbb {D}}}}{2}\chi \right) , \end{aligned}$$
  10 $$\begin{aligned} {\mathcal {H}}_{3}=-\frac{\gamma _{2}}{2 \gamma _{3}}+\frac{\sqrt{-{\mathbb {D}}}}{2\gamma _{3}}\left( \tan _{a}\left( \sqrt{-{\mathbb {D}}}\chi \right) \pm \sqrt{mn}\sec _{a}\left( \sqrt{-{\mathbb {D}}\chi }\right)\right).  \end{aligned}$$
  11 $$\begin{aligned} {\mathcal {H}}_{4}=-\frac{\gamma _{2}}{2 \gamma _{3}}+\frac{\sqrt{-{\mathbb {D}}}}{2\gamma _{3}}\left( \cot _{a}\left( \sqrt{-{\mathbb {D}}}\chi \right) \pm \sqrt{mn}\csc _{a}\left( \sqrt{-{\mathbb {D}}\chi }\right) \right), \end{aligned}$$
  12 $$\begin{aligned} {\mathcal {H}}_{5}=-\frac{\gamma _{2}}{2 \gamma _{3}}+\frac{\sqrt{-{\mathbb {D}}}}{2\gamma _{3}}\left( \tan _{a}\left( \frac{\sqrt{-{\mathbb {D}}}}{4}\chi \right) -\cot _{a} \left( \frac{\sqrt{-{\mathbb {D}}}}{4}\chi\right) \right) . \end{aligned}$$
- (2): If $$\gamma _2^2-4 \gamma _1 \gamma _3>0$$, and $$\gamma _3 \ne 0$$,
  13 $$\begin{aligned} {\mathcal {H}}_{6}= & -\frac{\gamma _{2}}{2 \gamma _{3}}-\frac{\sqrt{{\mathbb {D}}}}{2\gamma _{3}} \tanh _{a}\left( \frac{\sqrt{{\mathbb {D}}}}{2}\chi \right) , \end{aligned}$$
  14 $$\begin{aligned} {\mathcal {H}}_{7}= & -\frac{\gamma _{2}}{2 \gamma _{3}}-\frac{\sqrt{{\mathbb {D}}}}{2\gamma _{3}} \coth _{a}\left( \frac{\sqrt{{\mathbb {D}}}}{2}\chi \right) , \end{aligned}$$
  15 $$\begin{aligned} {\mathcal {H}}_{8}= & -\frac{\gamma _{2}}{2 \gamma _{3}}+\frac{\sqrt{{\mathbb {D}}}}{2\gamma _{3}} \left( -\tanh _{a}\left( \sqrt{{\mathbb {D}}}\chi \right) \pm i \sqrt{mn}sech_{a}\left( \sqrt{{\mathbb {D}}\chi }\right) \right) , \end{aligned}$$
  16 $$\begin{aligned} {\mathcal {H}}_{9}= & -\frac{\gamma _{2}}{2 \gamma _{3}}+\frac{\sqrt{{\mathbb {D}}}}{2\gamma _{3}} \left( -\coth _{a}\left( \sqrt{{\mathbb {D}}}\chi \right) \pm \sqrt{mn}csch_{a}\left( \sqrt{{\mathbb {D}}}\chi \right) \right) , \end{aligned}$$
  17 $$\begin{aligned} {\mathcal {H}}_{10}= & -\frac{\gamma _{2}}{2 \gamma _{3}}-\frac{\sqrt{{\mathbb {D}}}}{2\gamma _{3}} \left( \tanh _{a}\left( \frac{\sqrt{{\mathbb {D}}}}{4}\chi \right) +\coth _{a}\left( \frac{\sqrt{{\mathbb {D}}}}{4}\chi \right) \right) . \end{aligned}$$
- (3): If $$\gamma _1 \gamma _3>0$$, and $$\gamma _3 = 0$$,
  18 $$\begin{aligned} {\mathcal {H}}_{11}= & \sqrt{\frac{\gamma _{1}}{\gamma _{3}}}\tan _{a}\left( \sqrt{\gamma _{1}\gamma _{3}}\chi \right) , \end{aligned}$$
  19 $$\begin{aligned} {\mathcal {H}}_{12}= & -\sqrt{\frac{\gamma _{1}}{\gamma _{3}}}\cot _{a}\left( \sqrt{\gamma _{1}\gamma _{3}}\chi \right) , \end{aligned}$$
  20 $$\begin{aligned} {\mathcal {H}}_{13}= & \sqrt{\frac{\gamma _{1}}{\gamma _{3}}}\left( \tan _{a}\left( 2\sqrt{\gamma _{1}\gamma _{3}}\chi \right) \pm \sqrt{mn}\sec _{a}\left( 2\sqrt{\gamma _{1}\gamma _{3}}\chi \right) \right) , \end{aligned}$$
  21 $$\begin{aligned} {\mathcal {H}}_{14}= & \sqrt{\frac{\gamma _{1}}{\gamma _{3}}}\left( -\cot _{a}\left( 2\sqrt{\gamma _{1}\gamma _{3}}\chi \right) \pm \sqrt{mn}\csc _{a}\left( 2\sqrt{\gamma _{1}\gamma _{3}}\chi \right) \right) , \end{aligned}$$
  22 $$\begin{aligned} {\mathcal {H}}_{15}= & \frac{1}{2}\sqrt{\frac{\gamma _{1}}{\gamma _{3}}}\left( tan_{a}\left( \frac{\sqrt{\gamma _{1}\gamma _{3}}}{2}\chi \right) -cot_{a}\left( \frac{\sqrt{\gamma _{1}\gamma _{3}}}{2}\chi \right) \right) . \end{aligned}$$
- (4): If $$\gamma _1 \gamma _3<0$$, and $$\gamma _3 = 0$$,
  23 $$\begin{aligned} {\mathcal {H}}_{16}= & -\sqrt{-\frac{\gamma _{1}}{\gamma _{3}}}\tanh _{a}\left( \sqrt{-\gamma _{1}\gamma _{3}}\chi \right) , \end{aligned}$$
  24 $$\begin{aligned} {\mathcal {H}}_{17}= & -\sqrt{-\frac{\gamma _{1}}{\gamma _{3}}}\coth _{a}\left( \sqrt{-\gamma _{1}\gamma _{3}}\chi \right) , \end{aligned}$$
  25 $$\begin{aligned} {\mathcal {H}}_{18}= & \sqrt{-\frac{\gamma _{1}}{\gamma _{3}}}\left( -\tanh _{a}\left( 2\sqrt{-\gamma _{1}\gamma _{3}}\chi \right) \pm i\sqrt{mn}sech_{a}\left( 2\sqrt{-\gamma _{1}\gamma _{3}}\chi \right) \right) , \end{aligned}$$
  26 $$\begin{aligned} {\mathcal {H}}_{19}= & \sqrt{-\frac{\gamma _{1}}{\gamma _{3}}}\left( -\coth _{a}\left( 2\sqrt{-\gamma _{1}\gamma _{3}}\chi \right) \pm \sqrt{mn}csch_{a}\left( 2\sqrt{-\gamma _{1}\gamma _{3}}\chi \right) \right) , \end{aligned}$$
  27 $$\begin{aligned} {\mathcal {H}}_{20}= & -\frac{1}{2}\sqrt{-\frac{\gamma _{1}}{\gamma _{3}}}\left( tan_{a}\left( \frac{-\sqrt{\gamma _{1}\gamma _{3}}}{2}\chi \right) -cot_{a}\left( \frac{-\sqrt{\gamma _{1}\gamma _{3}}}{2}\chi \right) \right) . \end{aligned}$$
- (5): If $$\gamma _2=0$$, and $$\gamma _3 = \gamma _{1}$$,
  28 $$\begin{aligned} {\mathcal {H}}_{21}= & \tan _{a}(\gamma _{1}\chi ), \end{aligned}$$
  29 $$\begin{aligned} {\mathcal {H}}_{22}= & -\cot _{a}(\gamma _{1}\chi ), \end{aligned}$$
  30 $$\begin{aligned} {\mathcal {H}}_{23}= & tan_{a}(2\gamma _{1}\chi )\pm \sqrt{mn}sec_{a}(2\gamma _{1}\chi ), \end{aligned}$$
  31 $$\begin{aligned} {\mathcal {H}}_{24}= & -cot_{a}(2\gamma _{1}\chi )\pm \sqrt{mn}csc_{a}(2\gamma _{1}\chi ), \end{aligned}$$
  32 $$\begin{aligned} {\mathcal {H}}_{25}= & \frac{1}{2}\left( \tan _{a}\left( \frac{\gamma _{1}}{2}\chi \right) -\cot _{a}\left( \frac{\gamma _{1}}{2\chi }\right) \right) . \end{aligned}$$
- (6): If $$\gamma _2=0$$, and $$\gamma _3 = -\gamma _{1}$$,
  33 $$\begin{aligned} {\mathcal {H}}_{26}= & -\tanh _{a}(\gamma _{1}\chi ), \end{aligned}$$
  34 $$\begin{aligned} {\mathcal {H}}_{27}= & -\coth _{a}(\gamma _{1}\chi ), \end{aligned}$$
  35 $$\begin{aligned} {\mathcal {H}}_{28}= & tanh_{a}(2\gamma _{1}\chi )\pm i\sqrt{mn}sech_{a}(2\gamma _{1}\chi ), \end{aligned}$$
  36 $$\begin{aligned} {\mathcal {H}}_{29}= & -coth_{a}(2\gamma _{1}\chi )\pm \sqrt{mn}csch_{a}(2\gamma _{1}\chi ), \end{aligned}$$
  37 $$\begin{aligned} {\mathcal {H}}_{30}= & -\frac{1}{2}\left( \tanh _{a}\left( \frac{\gamma _{1}}{2}\chi \right) -\coth _{a}\left( \frac{\gamma _{1}}{2}\chi \right) \right) . \end{aligned}$$
- (7): If $$\gamma _2^{2}=4\gamma _{1}\gamma _{3}$$,
  38 $$\begin{aligned} {\mathcal {H}}_{31}=\frac{-2\gamma _{1}(\gamma _{2}\chi ln(a)+2)}{\gamma _{2}^{2}\chi ln(a)}. \end{aligned}$$
  In this case, *n* is used by rewriting the balancing principle using Eq. (5). The determining equation system is obtained by equating the coefficients of each power of ${\mathcal {H}}(\chi )$ to zero and then inserting Eqs. (6) and (7) into Eq. (5). Different values of $\alpha _i,~\kappa ,~\delta$ and $\vartheta$ are obtained by solving the obtained system, and these values will be used to find the solution of Eq. (3).

## Applications of NEDAM

In this section, we use the NEDAM to achieve the widely used explicit optical soliton solutions to the model under consideration. In particular, we carry out the subsequent wave transformations:

39 $$\begin{aligned} \Theta ( x,t)= & \Omega (\chi )e^{i\phi }(x,t), \end{aligned}$$

$$\chi =\frac{\Gamma (\beta +1) \left( x^{\alpha }-\vartheta t^{\alpha }\right) }{\alpha },~\phi =-\frac{\Gamma (\beta +1) \left( \kappa x^{\alpha }-\delta \vartheta t^{\alpha }\right) }{\alpha }.$$

Where $\kappa$ and $\vartheta$ refers as speed and frequency of the soliton respectively. Switching Eq. (39) into Eq. (1), Eq. (1) split into real and imaginary components:

40 $$\begin{aligned} {\left\{ \begin{array}{ll} Re:~ (-\delta -\kappa )\Omega +3 \kappa \Omega ^{''}+(\wp _{1}+\wp _{2}\kappa )\Omega ^{3}=0,\\ Im:~ \Omega ^{'''}-(3\kappa ^{2}+ \vartheta )\Omega ^{'}+(\wp _{2}+2\wp _{3})\Omega ^{2}\Omega ^{'}=0, \end{array}\right. } \end{aligned}$$

Integrate the Eq. (40) and setting the integral’s constant to zero, we push

41 $$\begin{aligned} \Omega ^{''}-(3\kappa ^{2}+ \vartheta )\Omega +\frac{1}{3}(\wp _{2}+2\wp _{3})\Omega ^{3}=0, \end{aligned}$$

Now, after reducing the imaginary and real parts of Eq. (40), we get:

42 $$\begin{aligned} \kappa= & \frac{1}{3},\nonumber \\ \vartheta= & \delta ,\nonumber \\ 3 \wp _{1}= & 2 \wp _{3}. \end{aligned}$$

Hence, in the rest of the work, we will consider the equation given below.

43 $$\begin{aligned} \Omega ^{''}-(\frac{1}{3}+\vartheta )\Omega +\frac{1}{3}(\wp _{2}+2\wp _{3})\Omega ^{3}=0, \end{aligned}$$

By establishing the balancing principle technique between the highest derivative $\Omega ^{''}$ with the highest power nonlinear term $\Omega ^{3}$ on Eq. (43), turns into $N = 1$. As a result, the nontrivial Eq. (43) becomes solution:

44 $$\begin{aligned} \Omega (\chi )= \alpha _0+\alpha _1 {\mathcal {H}}(\chi ). \end{aligned}$$

Putting (44) togather with its derivatives in (43), and collect all the coeffcients of the same of power of $\Omega (\chi )$ to get the system of equations, after solving the equations obtained the solution sets, which are described in below:

$${\textbf {Family-1:}}$$

45 $$\begin{aligned} {\left\{ \begin{array}{ll} \alpha _0\rightarrow \frac{\sqrt{\frac{3}{2}} \gamma _2 \text {Ln}(a)}{\sqrt{-\wp _2-2 \wp _3}},~\alpha _1\rightarrow \frac{\sqrt{6} \gamma _3 \text {Ln}(a)}{\sqrt{-\wp _2-2 \wp _3}},~\vartheta \rightarrow -\frac{1}{2} \left( \gamma _2^2-4 \gamma _1 \gamma _3\right) \text {Ln}(a)^2-\frac{1}{3}. \end{array}\right. } \end{aligned}$$

We obtain scores for the subsequent answers to Eq. (1):

(i) For $${\mathbb {D}}=\gamma _2^2-4 \gamma _1 \gamma _3<0$$, and $$\gamma _3 \ne 0$$.
  The trigonometric form solutions:
    **Cluster 1:**
    46 $$\begin{aligned} \Theta _{1}(x,t)=\left[ \frac{\sqrt{\frac{3}{2}} \sqrt{-{\mathbb {D}}} \text {Ln}(a) \text {Tan}_a\left( \frac{\sqrt{-{\mathbb {D}}} \chi }{2}\right) }{\sqrt{-\wp _2-2 \wp _3}}\right] \times e^{ \left( -\frac{i \Gamma (\beta +1) \left( 3 \left( \gamma _2^2-4 \gamma _1 \gamma _3\right) \delta \text {Ln}(a)^2 t^{\alpha }+2 \delta t^{\alpha }+6 \kappa x^{\alpha }\right) }{6 \alpha }\right) }. \end{aligned}$$
    **Cluster 2:**
    47 $$\begin{aligned} \Theta _{2}(x,t)=\left[ \frac{\sqrt{\frac{3}{2}} \sqrt{-{\mathbb {D}}} \text {Ln}(a) \text {Cot}_a\left( \frac{\sqrt{-{\mathbb {D}}} \chi }{2}\right) }{\sqrt{-\wp _2-2 \wp _3}}\right] \times e^{ \left( -\frac{i \Gamma (\beta +1) \left( 3 \left( \gamma _2^2-4 \gamma _1 \gamma _3\right) \delta \text {Ln}(a)^2 t^{\alpha }+2 \delta t^{\alpha }+6 \kappa x^{\alpha }\right) }{6 \alpha }\right) }. \end{aligned}$$
    The combo-trigonometric solutions:
    **Cluster 3:**
    48 $$\begin{aligned} \Theta _{3}(x,t)= & \left[ \frac{\sqrt{\frac{3}{2}} \sqrt{-{\mathbb {D}}} \text {Ln}(a) \left( \sqrt{m n} \text {Sec}_a\left( \sqrt{-{\mathbb {D}}} \chi \right) +\text {Tan}_a\left( \sqrt{-{\mathbb {D}}} \chi \right) \right) }{\sqrt{-\wp _2-2 \wp _3}}\right] \nonumber \\ & \times e^{ \left( -\frac{i \Gamma (\beta +1) \left( 3 \left( \gamma _2^2-4 \gamma _1 \gamma _3\right) \delta \text {Ln}(a)^2 t^{\alpha }+2 \delta t^{\alpha }+6 \kappa x^{\alpha }\right) }{6 \alpha }\right) }. \end{aligned}$$
    **Cluster 4:**
    49 $$\begin{aligned} \Theta _{4}(x,t)= & \left[ \frac{\sqrt{\frac{3}{2}} \sqrt{-{\mathbb {D}}} \text {Ln}(a) \left( \text {Cot}_a\left( \sqrt{-{\mathbb {D}}} \chi \right) +\sqrt{m n} \text {Csc}_a\left( \sqrt{-{\mathbb {D}}} \chi \right) \right) }{\sqrt{-\wp _2-2 \wp _3}}\right] \nonumber \\ & \times e^{ \left( -\frac{i \Gamma (\beta +1) \left( 3 \left( \gamma _2^2-4 \gamma _1 \gamma _3\right) \delta \text {Ln}(a)^2 t^{\alpha }+2 \delta t^{\alpha }+6 \kappa x^{\alpha }\right) }{6 \alpha }\right) }. \end{aligned}$$
    **Cluster 5:**
    50 $$\begin{aligned} \Theta _{5}(x,t)= & \left[ -\frac{\sqrt{\frac{3}{2}} \sqrt{-{\mathbb {D}}} \text {Ln}(a) \left( \text {Cot}_a\left( \sqrt{-{\mathbb {D}}} \chi \right) -\text {Tan}_a\left( \sqrt{-{\mathbb {D}}} \chi \right) \right) }{\sqrt{-\wp _2-2 \wp _3}}\right] \nonumber \\ & \times e^{ \left( -\frac{i \Gamma (\beta +1) \left( 3 \left( \gamma _2^2-4 \gamma _1 \gamma _3\right) \delta \text {Ln}(a)^2 t^{\alpha }+2 \delta t^{\alpha }+6 \kappa x^{\alpha }\right) }{6 \alpha }\right) }. \end{aligned}$$
(2) For $${\mathbb {D}}=\gamma _2^2-4 \gamma _1 \gamma _3>0$$, and $$\gamma _3 \ne 0$$
  The dark optical solution:
    **Cluster 6:**
    51 $$\begin{aligned} \Theta _{6}(x,t)= & \left[ -\frac{\sqrt{\frac{3}{2}} \sqrt{{\mathbb {D}}} \text {Ln}(a) \text {Tanh}_a\left( \frac{\sqrt{{\mathbb {D}}} \chi }{2}\right) }{\sqrt{-\wp _2-2 \wp _3}}\right] \nonumber \\ & \times e^{\left( -\frac{i \Gamma (\beta +1) \left( 3 \left( \gamma _2^2-4 \gamma _1 \gamma _3\right) \delta \text {Ln}(a)^2 t^{\alpha }+2 \delta t^{\alpha }+6 \kappa x^{\alpha }\right) }{6 \alpha }\right) }. \end{aligned}$$
    **Cluster 7:**
    52 $$\begin{aligned} \Theta _{7}(x,t)= & \left[ -\frac{\sqrt{\frac{3}{2}} \sqrt{{\mathbb {D}}} \text {Ln}(a) \text {Coth}_a\left( \frac{\sqrt{{\mathbb {D}}} \chi }{2}\right) }{\sqrt{-\wp _2-2 \wp _3}}\right] \nonumber \\ & \times e^{ \left( -\frac{i \Gamma (\beta +1) \left( 3 \left( \gamma _2^2-4 \gamma _1 \gamma _3\right) \delta \text {Ln}(a)^2 t^{\alpha }+2 \delta t^{\alpha }+6 \kappa x^{\alpha }\right) }{6 \alpha }\right) }. \end{aligned}$$
  The bright–dark optical soliton solution:
    **Cluster 8:**
    53 $$\begin{aligned} \Theta _{8}(x,t)= & \left[ \frac{i \sqrt{\frac{3}{2}} \sqrt{{\mathbb {D}}} \text {Ln}(a) \left( \sqrt{m n} \text {Sech}_a\left( \sqrt{{\mathbb {D}}} \chi \right) +i \text {Tanh}_a\left( \sqrt{{\mathbb {D}}} \chi \right) \right) }{\sqrt{-\wp _2-2 \wp _3}}\right] \nonumber \\ & \times e^{ \left( -\frac{i \Gamma (\beta +1) \left( 3 \left( \gamma _2^2-4 \gamma _1 \gamma _3\right) \delta \text {Ln}(a)^2 t^{\alpha }+2 \delta t^{\alpha }+6 \kappa x^{\alpha }\right) }{6 \alpha }\right) }. \end{aligned}$$
  The solution for mixed singular optical soliton:
    **Cluster 9:**
    54 $$\begin{aligned} \Theta _{9}(x,t)= & \left[ -\frac{\sqrt{\frac{3}{2}} \sqrt{{\mathbb {D}}} \text {Ln}(a) \left( \text {Coth}_a\left( \sqrt{{\mathbb {D}}} \chi \right) -\sqrt{m n} \text {Csch}_a\left( \sqrt{{\mathbb {D}}} \chi \right) \right) }{\sqrt{-\wp _2-2 \wp _3}}\right] \nonumber \\ & \times e^{ \left( -\frac{i \Gamma (\beta +1) \left( 3 \left( \gamma _2^2-4 \gamma _1 \gamma _3\right) \delta \text {Ln}(a)^2 t^{\alpha }+2 \delta t^{\alpha }+6 \kappa x^{\alpha }\right) }{6 \alpha }\right) }. \end{aligned}$$
  The intricate optical soliton solution at dark-singularity:
    **Cluster 10:**
    55 $$\begin{aligned} \Theta _{10}(x,t)= & \left[ -\frac{\sqrt{\frac{3}{2}} \sqrt{{\mathbb {D}}} \text {Ln}(a) \left( \text {Coth}_a\left( \sqrt{{\mathbb {D}}} \chi \right) -\sqrt{m n} \text {Csch}_a\left( \sqrt{{\mathbb {D}}} \chi \right) \right) }{\sqrt{-\wp _2-2 \wp _3}}\right] \nonumber \\ & \times e^{ \left( -\frac{i \Gamma (\beta +1) \left( 3 \left( \gamma _2^2-4 \gamma _1 \gamma _3\right) \delta \text {Ln}(a)^2 t^{\alpha }+2 \delta t^{\alpha }+6 \kappa x^{\alpha }\right) }{6 \alpha }\right) }. \end{aligned}.$$
(3) For $$\gamma _1 \gamma _3>0$$, and $$\gamma _3 = 0$$
  The periodic solutions:
    **Cluster 11:**
    56 $$\begin{aligned} \Theta _{11}(x,t)= & \left[ \frac{\sqrt{\frac{3}{2}} \text {Ln}(a) \left( \gamma _2-2 \sqrt{\frac{\gamma _1}{\gamma _3}} \gamma _3 \text {Tan}_a\left( \sqrt{\gamma _1 \gamma _3} \chi \right) \right) }{\sqrt{-\wp _2-2 \wp _3}}\right] \nonumber \\ & \times e^{ \left( -\frac{i \Gamma (\beta +1) \left( 3 \left( \gamma _2^2-4 \gamma _1 \gamma _3\right) \delta \text {Ln}(a)^2 t^{\alpha }+2 \delta t^{\alpha }+6 \kappa x^{\alpha }\right) }{6 \alpha }\right) }. \end{aligned}$$
    **Cluster 12:**
    57 $$\begin{aligned} \Theta _{12}(x,t)= & \left[ \frac{\sqrt{\frac{3}{2}} \text {Ln}(a) \left( \gamma _2-2 \sqrt{\frac{\gamma _1}{\gamma _3}} \gamma _3 \text {Cot}_a\left( \sqrt{\gamma _1 \gamma _3} \chi \right) \right) }{\sqrt{-\wp _2-2 \wp _3}}\right] \nonumber \\ & \times e^{ \left( -\frac{i \Gamma (\beta +1) \left( 3 \left( \gamma _2^2-4 \gamma _1 \gamma _3\right) \delta \text {Ln}(a)^2 t^{\alpha }+2 \delta t^{\alpha }+6 \kappa x^{\alpha }\right) }{6 \alpha }\right) }. \end{aligned}$$
  The mixed trigonometric solutions:
    **Cluster 13:**
    58 $$\begin{aligned} \Theta _{13}(x,t)= & \left[ \frac{\sqrt{\frac{3}{2}} \text {Ln}(a) \left( 2 \sqrt{\frac{\gamma _1}{\gamma _3}} \gamma _3 \left( \sqrt{m n} \text {Sec}_a\left( 2 \sqrt{\gamma _1 \gamma _3} \chi \right) +\text {Tan}_a\left( 2 \sqrt{\gamma _1 \gamma _3} \chi \right) \right) +\gamma _2\right) }{\sqrt{-\wp _2-2 \wp _3}}\right] \nonumber \\ & \times e^{ \left( -\frac{i \Gamma (\beta +1) \left( 3 \left( \gamma _2^2-4 \gamma _1 \gamma _3\right) \delta \text {Ln}(a)^2 t^{\alpha }+2 \delta t^{\alpha }+6 \kappa x^{\alpha }\right) }{6 \alpha }\right) }. \end{aligned}$$
    **Cluster 14:**
    59 $$\begin{aligned} \Theta _{14}(x,t)= & \left[ \frac{\sqrt{\frac{3}{2}} \text {Ln}(a) \left( \gamma _2-2 \sqrt{\frac{\gamma _1}{\gamma _3}} \gamma _3 \left( \text {Cot}_a\left( 2 \sqrt{\gamma _1 \gamma _3} \chi \right) -\sqrt{m n} \text {Csc}_a\left( 2 \sqrt{\gamma _1 \gamma _3} \chi \right) \right) \right) }{\sqrt{-\wp _2-2 \wp _3}}\right] \nonumber \\ & \times e^{ \left( -\frac{i \Gamma (\beta +1) \left( 3 \left( \gamma _2^2-4 \gamma _1 \gamma _3\right) \delta \text {Ln}(a)^2 t^{\alpha }+2 \delta t^{\alpha }+6 \kappa x^{\alpha }\right) }{6 \alpha }\right) }. \end{aligned}$$
    **Cluster 15:**
    60 $$\begin{aligned} \Theta _{15}(x,t)= & \left[ \frac{\sqrt{\frac{3}{2}} \text {Ln}(a) \left( \sqrt{\frac{\gamma _1}{\gamma _3}} \gamma _3 \left( \text {Tan}_a\left( \frac{1}{2} \sqrt{\gamma _1 \gamma _3} \chi \right) -\text {Cot}_a\left( \frac{1}{2} \sqrt{\gamma _1 \gamma _3} \chi \right) \right) +\gamma _2\right) }{\sqrt{-\wp _2-2 \wp _3}}\right] \nonumber \\ & \times e^{ \left( -\frac{i \Gamma (\beta +1) \left( 3 \left( \gamma _2^2-4 \gamma _1 \gamma _3\right) \delta \text {Ln}(a)^2 t^{\alpha }+2 \delta t^{\alpha }+6 \kappa x^{\alpha }\right) }{6 \alpha }\right) }. \end{aligned}$$.
(4) For $$\gamma _1 \gamma _3<0$$, and $$\gamma _3 = 0$$
  The dark optical solutions:
    **Cluster 16:**
    61 $$\begin{aligned} \Theta _{16}(x,t)= & \left[ \frac{\sqrt{\frac{3}{2}} \text {Ln}(a) \left( \gamma _2-2 \sqrt{-\frac{\gamma _1}{\gamma _3}} \gamma _3 \text {Tanh}_a\left( \sqrt{-\gamma _1 \gamma _3} \chi \right) \right) }{\sqrt{-\wp _2-2 \wp _3}}\right] \nonumber \\ & \times e^{ \left( -\frac{i \Gamma (\beta +1) \left( 3 \left( \gamma _2^2-4 \gamma _1 \gamma _3\right) \delta \text {Ln}(a)^2 t^{\alpha }+2 \delta t^{\alpha }+6 \kappa x^{\alpha }\right) }{6 \alpha }\right) }. \end{aligned}$$
  The singular optical solution:
    **Cluster 17:**
    62 $$\begin{aligned} \Theta _{17}(x,t)= & \left[ \frac{\sqrt{\frac{3}{2}} \text {Ln}(a) \left( \gamma _2-2 \sqrt{-\frac{\gamma _1}{\gamma _3}} \gamma _3 \text {Coth}_a\left( \sqrt{-\gamma _1 \gamma _3} \chi \right) \right) }{\sqrt{-\wp _2-2 \wp _3}}\right] \nonumber \\ & \times e^{ \left( -\frac{i \Gamma (\beta +1) \left( 3 \left( \gamma _2^2-4 \gamma _1 \gamma _3\right) \delta \text {Ln}(a)^2 t^{\alpha }+2 \delta t^{\alpha }+6 \kappa x^{\alpha }\right) }{6 \alpha }\right) }. \end{aligned}$$
  Complexiton mixed type solutions:
    **Cluster 18:**
    63 $$\begin{aligned} \Theta _{18}(x,t)= & \left[ \frac{\sqrt{\frac{3}{2}} \text {Ln}(a) \left( \gamma _2+2 i \sqrt{-\frac{\gamma _1}{\gamma _3}} \gamma _3 \left( \sqrt{m n} \text {Sech}_a\left( 2 \sqrt{-\gamma _1 \gamma _3} \chi \right) +i \text {Tanh}_a\left( 2 \sqrt{-\gamma _1 \gamma _3} \chi \right) \right) \right) }{\sqrt{-\wp _2-2 \wp _3}}\right] \nonumber \\ & \times e^{ \left( -\frac{i \Gamma (\beta +1) \left( 3 \left( \gamma _2^2-4 \gamma _1 \gamma _3\right) \delta \text {Ln}(a)^2 t^{\alpha }+2 \delta t^{\alpha }+6 \kappa x^{\alpha }\right) }{6 \alpha }\right) }. \end{aligned}$$
    **Cluster 19:**
    64 $$\begin{aligned} \Theta _{19}(x,t)= & \left[ \frac{\sqrt{\frac{3}{2}} \text {Ln}(a) \left( \gamma _2-2 \sqrt{-\frac{\gamma _1}{\gamma _3}} \gamma _3 \left( \text {Coth}_a\left( 2 \sqrt{-\gamma _1 \gamma _3} \chi \right) -\sqrt{m n} \text {Csch}_a\left( 2 \sqrt{-\gamma _1 \gamma _3} \chi \right) \right) \right) }{\sqrt{-\wp _2-2 \wp _3}}\right] \nonumber \\ & \times e^{ \left( -\frac{i \Gamma (\beta +1) \left( 3 \left( \gamma _2^2-4 \gamma _1 \gamma _3\right) \delta \text {Ln}(a)^2 t^{\alpha }+2 \delta t^{\alpha }+6 \kappa x^{\alpha }\right) }{6 \alpha }\right) }. \end{aligned}$$
    **Cluster 20:**
    65 $$\begin{aligned} \Theta _{20}(x,t)= & \left[ \frac{\sqrt{\frac{3}{2}} \text {Ln}(a) \left( \frac{\gamma _1 \left( \text {Coth}_a\left( \sqrt{-\gamma _1 \gamma _3} \chi \right) +\text {Tanh}_a\left( \sqrt{-\gamma _1 \gamma _3} \chi \right) \right) }{\sqrt{-\frac{\gamma _1}{\gamma _3}}}+\gamma _2\right) }{\sqrt{-\wp _2-2 \wp _3}}\right] \nonumber \\ & \times e^{ \left( -\frac{i \Gamma (\beta +1) \left( 3 \left( \gamma _2^2-4 \gamma _1 \gamma _3\right) \delta \text {Ln}(a)^2 t^{\alpha }+2 \delta t^{\alpha }+6 \kappa x^{\alpha }\right) }{6 \alpha }\right) }. \end{aligned}$$.
(5) For $$\gamma _2=0$$, and $$\gamma _1 = \gamma _3$$
  The answers to periodic waves are as follows:
    **Cluster 21:**
    66 $$\begin{aligned} \Theta _{21}(x,t)= & \left[ \frac{\sqrt{\frac{3}{2}} \text {Ln}(a) \left( 2 \gamma _3 \text {Tan}_a\left( \gamma _1 \chi \right) +\gamma _2\right) }{\sqrt{-\wp _2-2 \wp _3}}\right] \nonumber \\ & \times e^{ \left( -\frac{i \Gamma (\beta +1) \left( 3 \left( \gamma _2^2-4 \gamma _1 \gamma _3\right) \delta \text {Ln}(a)^2 t^{\alpha }+2 \delta t^{\alpha }+6 \kappa x^{\alpha }\right) }{6 \alpha }\right) }. \end{aligned}$$
    **Cluster 22:**
    67 $$\begin{aligned} \Theta _{22}(x,t)=\left[ \frac{\sqrt{\frac{3}{2}} \text {Ln}(a) \left( \gamma _2-2 \gamma _3 \text {Cot}_a\left( \gamma _1 \chi \right) \right) }{\sqrt{-\wp _2-2 \wp _3}}\right] \times e^{ \left( -\frac{i \Gamma (\beta +1) \left( 3 \left( \gamma _2^2-4 \gamma _1 \gamma _3\right) \delta \text {Ln}(a)^2 t^{\alpha }+2 \delta t^{\alpha }+6 \kappa x^{\alpha }\right) }{6 \alpha }\right) }. \end{aligned}$$
  Combined trigonometric solutions:
    **Cluster 23:**
    68 $$\begin{aligned} \Theta _{23}(x,t)= & \left[ \frac{\sqrt{\frac{3}{2}} \text {Ln}(a) \left( 2 \gamma _3 \left( \sqrt{m n} \text {Sec}_a\left( 2 \gamma _1 \chi \right) +\text {Tan}_a\left( 2 \gamma _1 \chi \right) \right) +\gamma _2\right) }{\sqrt{-\wp _2-2 \wp _3}}\right] \nonumber \\ & \times e^{ \left( -\frac{i \Gamma (\beta +1) \left( 3 \left( \gamma _2^2-4 \gamma _1 \gamma _3\right) \delta \text {Ln}(a)^2 t^{\alpha }+2 \delta t^{\alpha }+6 \kappa x^{\alpha }\right) }{6 \alpha }\right) }. \end{aligned}$$
    **Cluster 24:**
    69 $$\begin{aligned} \Theta _{24}(x,t)= & \left[ \frac{\sqrt{\frac{3}{2}} \text {Ln}(a) \left( \gamma _2-2 \gamma _3 \left( \text {Cot}_a\left( 2 \gamma _1 \chi \right) -\sqrt{m n} \text {Csc}_a\left( 2 \gamma _1 \chi \right) \right) \right) }{\sqrt{-\wp _2-2 \wp _3}}\right] \nonumber \\ & \times e^{ \left( -\frac{i \Gamma (\beta +1) \left( 3 \left( \gamma _2^2-4 \gamma _1 \gamma _3\right) \delta \text {Ln}(a)^2 t^{\alpha }+2 \delta t^{\alpha }+6 \kappa x^{\alpha }\right) }{6 \alpha }\right) }. \end{aligned}$$
    **Cluster 25:**
    70 $$\begin{aligned} \Theta _{25}(x,t)= & \left[ \frac{\sqrt{\frac{3}{2}} \text {Ln}(a) \left( \gamma _3 \left( \text {Tan}_a\left( \frac{\gamma _1 \chi }{2}\right) -\text {Cot}_a\left( \frac{\gamma _1 \chi }{2}\right) \right) +\gamma _2\right) }{\sqrt{-\wp _2-2 \wp _3}}\right] \nonumber \\ & \times e^{ \left( -\frac{i \Gamma (\beta +1) \left( 3 \left( \gamma _2^2-4 \gamma _1 \gamma _3\right) \delta \text {Ln}(a)^2 t^{\alpha }+2 \delta t^{\alpha }+6 \kappa x^{\alpha }\right) }{6 \alpha }\right) }. \end{aligned}$$.
(6) For $$\gamma _2=0$$, and $$\gamma _1 =- \gamma _3$$
  Exact wave solutions:
    **Cluster 26:**
    71 $$\begin{aligned} \Theta _{26}(x,t)=\left[ \frac{\sqrt{\frac{3}{2}} \text {Ln}(a) \left( \gamma _2-2 \gamma _3 \text {Tanh}_a\left( \gamma _1 \chi \right) \right) }{\sqrt{-\wp _2-2 \wp _3}}\right] \times e^{ \left( -\frac{i \Gamma (\beta +1) \left( 3 \left( \gamma _2^2-4 \gamma _1 \gamma _3\right) \delta \text {Ln}(a)^2 t^{\alpha }+2 \delta t^{\alpha }+6 \kappa x^{\alpha }\right) }{6 \alpha }\right) }. \end{aligned}$$
    **Cluster 27:**
    72 $$\begin{aligned} \Theta _{27}(x,t)=\left[ \frac{\sqrt{\frac{3}{2}} \text {Ln}(a) \left( \gamma _2-2 \gamma _3 \text {Coth}_a\left( \gamma _1 \chi \right) \right) }{\sqrt{-\wp _2-2 \wp _3}}\right] \times e^{ \left( -\frac{i \Gamma (\beta +1) \left( 3 \left( \gamma _2^2-4 \gamma _1 \gamma _3\right) \delta \text {Ln}(a)^2 t^{\alpha }+2 \delta t^{\alpha }+6 \kappa x^{\alpha }\right) }{6 \alpha }\right) }. \end{aligned}$$
    **Cluster 28:**
    73 $$\begin{aligned} \Theta _{28}(x,t)= & \left[ \frac{\sqrt{\frac{3}{2}} \text {Ln}(a) \left( \gamma _2+2 i \gamma _3 \left( \sqrt{m n} \text {Sech}_a\left( 2 \gamma _1 \chi \right) +i \text {Tanh}_a\left( 2 \gamma _1 \chi \right) \right) \right) }{\sqrt{-\wp _2-2 \wp _3}}\right] \nonumber \\ & \times e^{ \left( -\frac{i \Gamma (\beta +1) \left( 3 \left( \gamma _2^2-4 \gamma _1 \gamma _3\right) \delta \text {Ln}(a)^2 t^{\alpha }+2 \delta t^{\alpha }+6 \kappa x^{\alpha }\right) }{6 \alpha }\right) }. \end{aligned}$$
    **Cluster 29:**
    74 $$\begin{aligned} \Theta _{29}(x,t)= & \left[ \frac{\sqrt{\frac{3}{2}} \text {Ln}(a) \left( \gamma _2-\gamma _3 \left( \text {Coth}_a\left( \frac{\gamma _1 \chi }{2}\right) +\text {Tanh}_a\left( \frac{\gamma _1 \chi }{2}\right) \right) \right) }{\sqrt{-\wp _2-2 \wp _3}}\right] \nonumber \\ & \times e^{ \left( -\frac{i \Gamma (\beta +1) \left( 3 \left( \gamma _2^2-4 \gamma _1 \gamma _3\right) \delta \text {Ln}(a)^2 t^{\alpha }+2 \delta t^{\alpha }+6 \kappa x^{\alpha }\right) }{6 \alpha }\right) }. \end{aligned}$$
    **Cluster 30:**
    75 $$\begin{aligned} \Theta _{30}(x,t)= & \left[ \frac{\sqrt{\frac{3}{2}} \left( \gamma _2 \left( \gamma _2^2-4 \gamma _1 \gamma _3\right) \chi \text {Ln}(a)-8 \gamma _1 \gamma _3\right) }{\gamma _2^2 \chi \sqrt{-\wp _2-2 \wp _3}}\right] \nonumber \\ & \times e^{ \left( -\frac{i \Gamma (\beta +1) \left( 3 \left( \gamma _2^2-4 \gamma _1 \gamma _3\right) \delta \text {Ln}(a)^2 t^{\alpha }+2 \delta t^{\alpha }+6 \kappa x^{\alpha }\right) }{6 \alpha }\right) }. \end{aligned}$$.
(7) For $$\gamma _2^{2}=4 \gamma _1 \gamma _3$$
  Rational function solution:
    **Cluster 31:**
    76 $$\begin{aligned} \Theta _{31}(x,t)=\left[ \frac{\sqrt{\frac{3}{2}} \left( \gamma _2 \chi \text {Ln}(a)-2\right) }{\chi \sqrt{-\wp _2-2 \wp _3}}\right] \times e^{ \left( -\frac{i \Gamma (\beta +1) \left( 3 \left( \gamma _2^2-4 \gamma _1 \gamma _3\right) \delta \text {Ln}(a)^2 t^{\alpha }+2 \delta t^{\alpha }+6 \kappa x^{\alpha }\right) }{6 \alpha }\right) }. \end{aligned}$$
    For above all solutions $\chi =\frac{\Gamma (\beta +1) \left( x^{\alpha }-t^{\alpha } \left( -\frac{1}{2} \left( \gamma _2^2-4 \gamma _1 \gamma _3\right) \text {Ln}(a)^2-\frac{1}{3}\right) \right) }{\alpha }$..

## Stability property

This section examines the Hamiltonian stability and properties of derived solitary wave solutions^34^ in order to shed light on the connection between mathematical and physical systems. The Hamiltonian property holds significant importance in the study of solitary wave solutions as it guarantees the existence and uniqueness of solutions, facilitates stability analysis, guides numerical methods, and provides a physical interpretation of the solutions. Calculating the momentum in Eq. (46), we obtain:

77 $$\begin{aligned} N=-3.8324 \left( \tan ^{-1}(\tan (0.61441 (\vartheta -3)))-\tan ^{-1}(\tan (0.61441 (\vartheta +3)))-\tan (0.61441 (\vartheta -3))+\tan (0.61441 (\vartheta +3))\right) . \end{aligned}$$

Therefore, the following ensures the stability of the studied model:

78 $$\begin{aligned} \frac{\partial {N}}{\partial {\vartheta }}|_{\vartheta =2.2}=0.668141>0. \end{aligned}$$

Thus, in $x \in ~[-3,3]$, Eq. (46) is a stable solution. The same method can be used to examine the stability conditions of different solutions.

## Bifurcation analysis

In this part, we systematically examine the bifurcation of the system^35^, which includes reviewing phase portraits for the system as specified by Eq. (79). Bifurcation analysis focuses on the qualitative changes in the behavior of a dynamical system as parameters are varied. Phase portraits provide visual representations of the behavior of dynamical systems in their phase space, which is typically the space of all possible states of the system. It provide insights into the stability of equilibrium points and periodic orbits. Stable points attract neighboring trajectories, while unstable points repel them. The dynamical system is as follow:

79 $$\begin{aligned} \left\{ \begin{array}{ll} \frac{d \Omega }{d\chi } ={\mathcal {W}},~~~~~~~~~~~~~~~& \\ \\ \frac{dW}{d\chi }=\mu _{1}\Omega (\chi )-\mu _{2}\Omega ^{3}(\chi ),& \end{array}\right. \end{aligned}$$

where $\mu _1=\vartheta +\frac{1}{3}$ and $\mu _2=\frac{1}{3} \left( \wp _2+2 \wp _3\right)$ such that $\vartheta ,~\wp _2,~\wp _3$ are parameters. The derived equilibrium points (EPs) of the system Eq. (79) are as follows:

$$\begin{aligned} \chi _{1}=(0,0),~~~~~~\chi _{2}=\left( \frac{\sqrt{\mu _1}}{\sqrt{\mu _2}},0\right) ,~~~~~~\chi _{3}=\left( -\frac{\sqrt{\mu _1}}{\sqrt{\mu _2}},0\right) . \end{aligned}$$

The Jacobian for the system (79) is:

80 $$\begin{aligned} {\mathcal {J}}(\Omega ,{\mathcal {W}})=\begin{vmatrix} 0&1 \\ \mu _1-3\mu _2\Omega ^2&0 \end{vmatrix}=\mu _1-3\mu _2\Omega ^2. \end{aligned}$$

Hence,

- ($\Omega$,0) denotes saddle if ${\mathcal {J}}(\Omega ,{\mathcal {W}}))<0$,
- ($\Omega$,0) denotes center if ${\mathcal {J}}(\Omega ,{\mathcal {W}})>0$,
- ($\Omega$,0) denotes cuspidal if ${\mathcal {J}}(\Omega ,{\mathcal {W}})=0$.

The following describes the possible results that can be obtained by changing the settings.

**Fig. 1:**
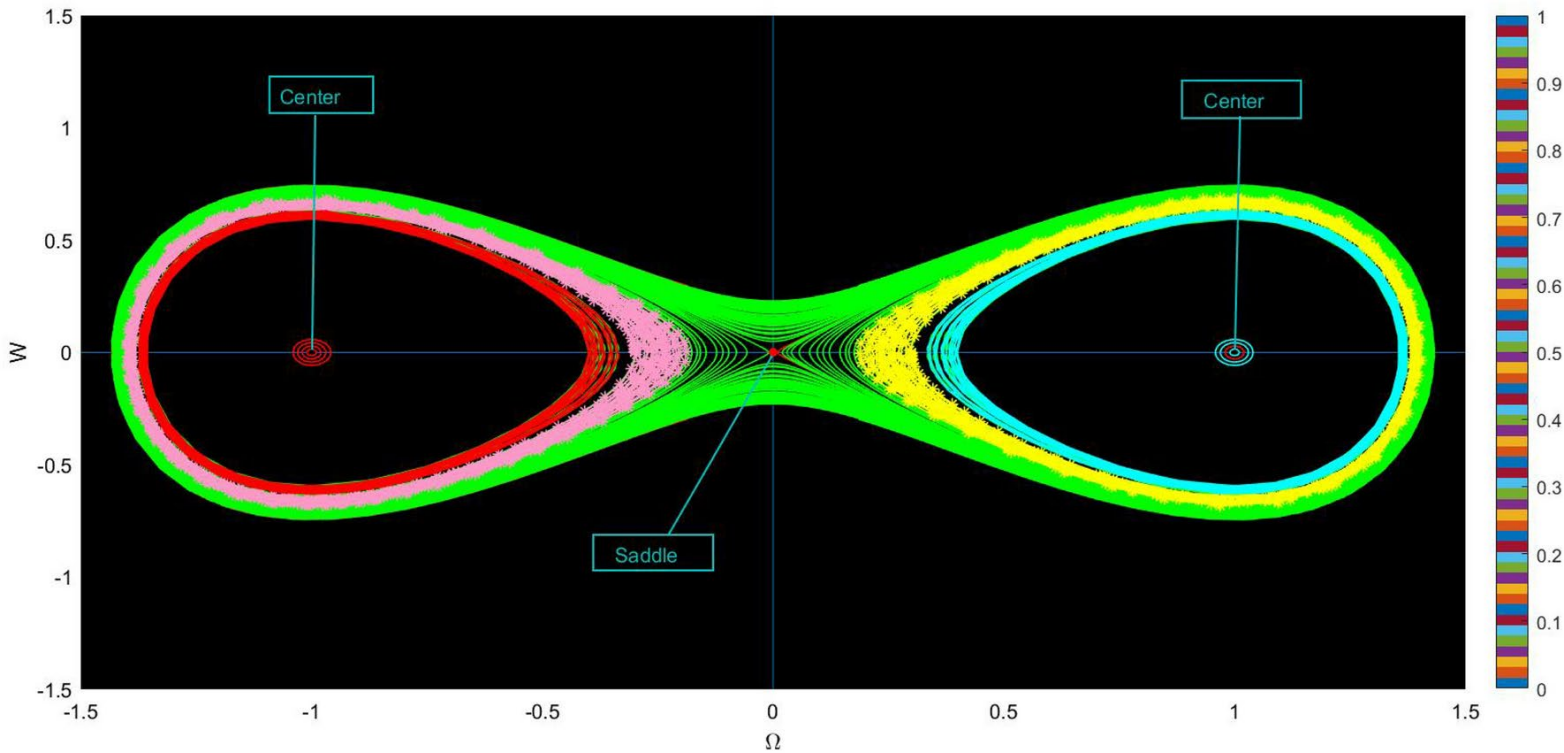
Physical visualization of phase variation plots of the proposed system of case (i).

**Fig. 2:**
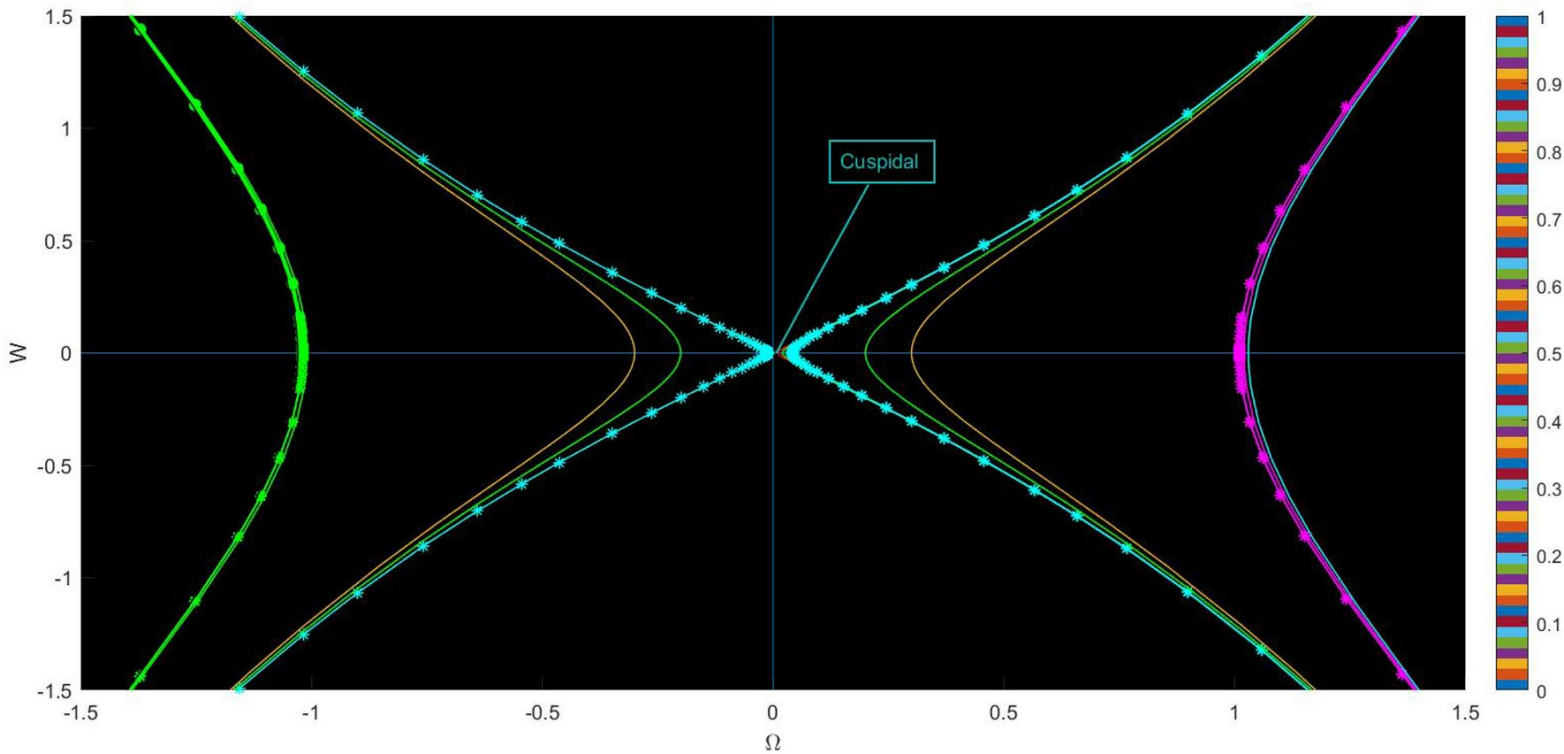
Physical visualization of phase variation plots of the proposed system of case (ii).

**Fig. 3:**
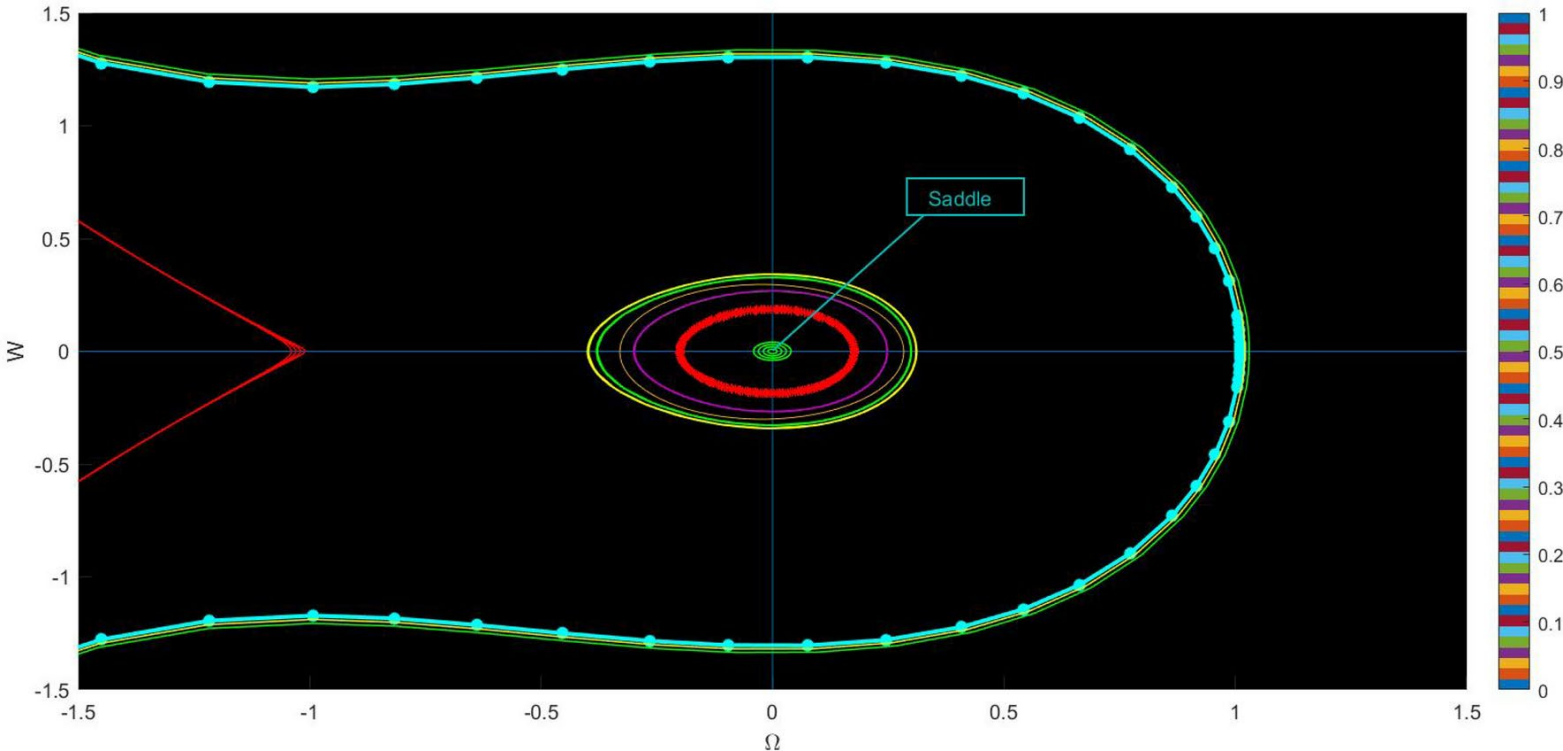
Physical visualization of phase variation plots of the proposed system of case (iii).

**Fig. 4:**
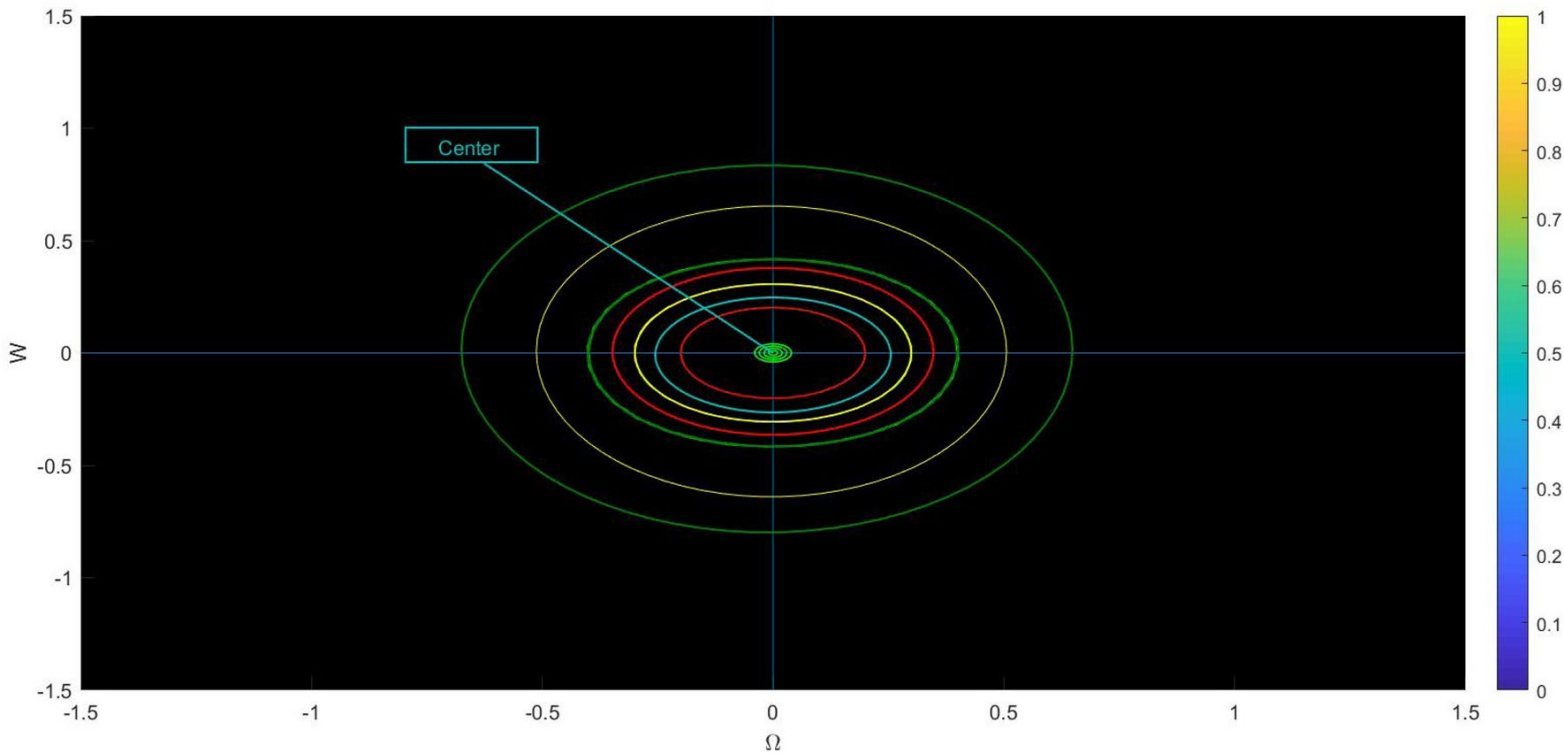
Physical visualization of phase variation plots of the proposed system of case (iv).

- **Case-(i)** When $\mu _{1}>0~ \& ~\mu _{2}>0$, under certain parameters, $\vartheta =.33,~ \wp _2=2.5 ~\text{ and }~ \wp _3 = 2.3$, we identified EPs, which are $(0,0),~(-1,0)~(1,0)$. These EPs are depicted in Fig. 1, with (0, 0) refers as a saddle point, while $(-1, 0)$ and (1, 0) represent center-like behavior.
- **Case-(ii)** When $\mu _{1}>0~ \& ~\mu _{2}<0$, upon applying specific parameter values, $\vartheta =-1,~ \wp _2=-1 ~\text{ and }~ \wp _3 = -3$, we find a single point (0, 0). This is represented in Fig. 2, with (0, 0) signifies the cusp point.
- **Case-(iii)** When $\mu _{1}<0~ \& ~\mu _{2}>0$, under certain parameters, $\vartheta =-1,~ \wp _2=3 ~\text{ and }~ \wp _3 = 5$, we identified three EPs. $(0,0),~(-1,0),~(1,0)$. These EPs are demonstrated in Fig. 3, where (0,0) signifies saddle point.
- **Case-(iv)** When $\mu _{1}<0~ \& ~\mu _{2}<0$, under certain parameters, $\vartheta =1.8,~ \wp _2=-0.2 ~\text{ and }~ \wp _3 = 0.06$, we identify EP: (0, 0). This EP is depicted in Fig. 4, which represent center-like behavior.

Bifurcation analysis and phase portraits offer powerful tools for understanding the behavior of complex dynamical systems, from simple mechanical systems to high-dimensional nonlinear models in various scientific disciplines. They provide both qualitative and quantitative insights into system behavior and serve as essential tools in theoretical and applied research.

### Chaotic structure of the model

Here, we study the chaotic behaviours shown by the model being investigated by introducing the additional perturbation term. We have included both 2D and 3D phase diagrams that are relevant to this system equation (81). Thus, the modified system depicted as follows:

81 $$\begin{aligned} \left\{ \begin{array}{ll} \frac{d \Omega }{d\chi } ={\mathcal {W}},~~~~~~~~~~~~~~~& \\ \\ \frac{dW}{d\chi }=\mu _{1}\Omega (\chi )-\mu _{2}\Omega ^{3}(\chi )+\omega _{1}\sin (\omega _{2}t).& \end{array}\right. \end{aligned}$$

**Fig. 5:**
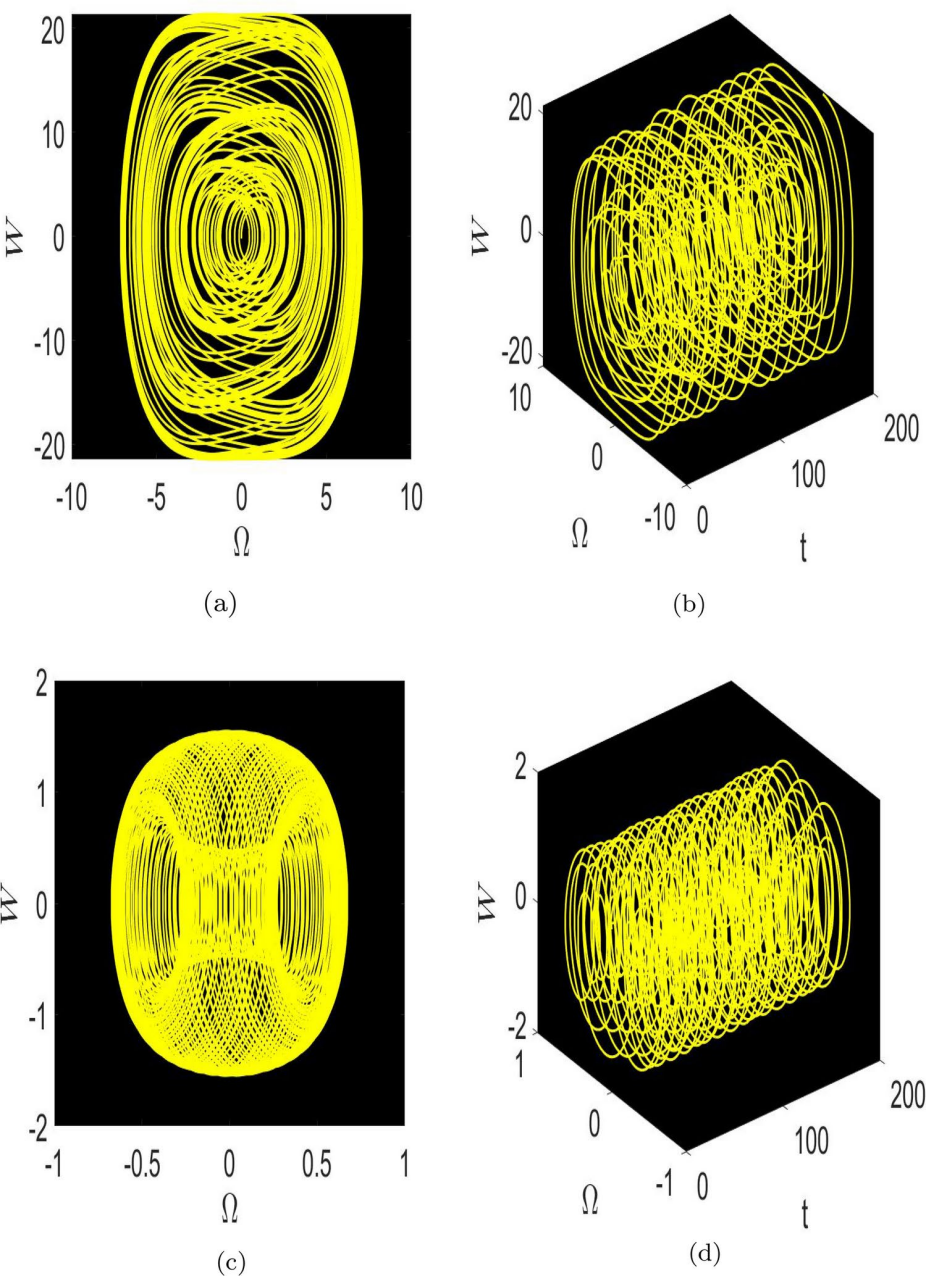
Physical visualization of 2D and 3D chaotic behavior of the system (81) with a arbitrary parametric values.

**Fig. 6:**
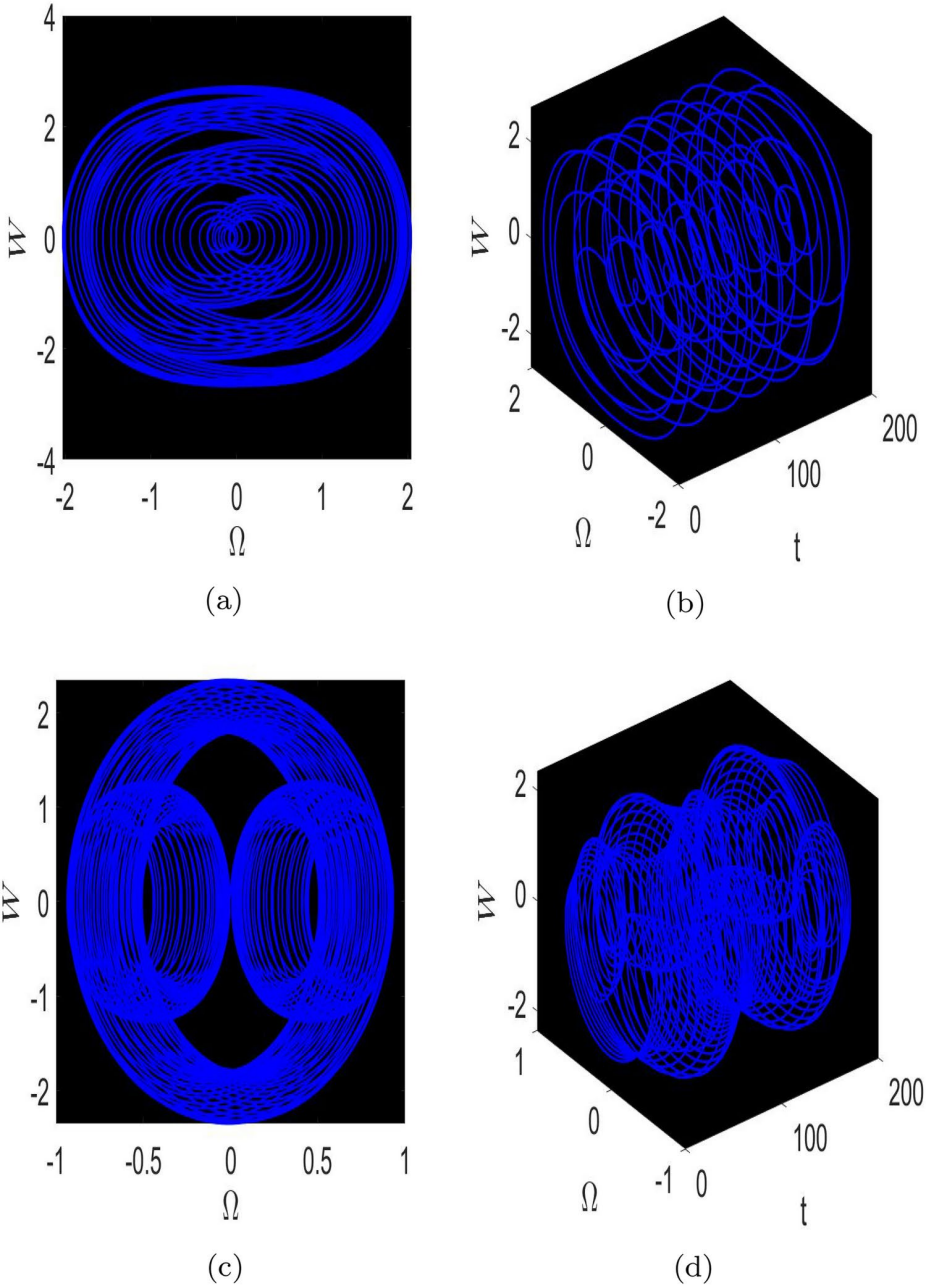
Physical visualization of 2D and 3D chaotic behavior of the system (81) with a arbitrary parametric values.

The dynamic behaviour of system Eq. (81) is seen by Figs. 5 and 6, which show the impact of perturbation term $\omega _{1}\sin (\omega _{2}t)$. Here, $\omega _{1}$ refers as the amplitude, while $\omega _{2}$ represents the frequency of the proposed system.

The 2D and 2D time phase diagrams for the system analysis are displayed using the following values: $\varphi =0.9,~\wp _1=0.5,~\wp _{2} =0.3$. Here, we take into consideration two sets of parameter combinations.: [(*a*), (*b*)] $\omega _{1}=2.5$, $\omega _{2}=8.91$, and [(*c*), (*d*)] $\omega _{1}=-5.1$, $\omega _{2}=4.91$, as visualized in Fig. 5. Additionally, in Fig. 6, we demonstrate two different sets of frequency and amplitude values: [(*a*), (*b*)] $\omega _{1}=5.2$, $\omega _{2} = 0.4$, and [(*c*), (*d*)] $\omega _{1}=3.5$, $\omega _{2}=4.2$, as shown in Fig. 6.

Following the phase portrait analysis, incredibly intricate and captivating dynamics are revealed. This demonstrates how the proposed system’s behaviour is susceptible to perturbations that develop in $\omega _{2}$. It also provides important insights into how the perturbation term $\omega _{1}sin(\omega _{2}t)$ affects the system’s behaviour. These new insights on the system’s susceptibility to changes in parameters improve our understanding of the intricate links between $\omega _{2}$ and the system’s dynamics as a whole. These insights effectively contribute to a more comprehensive comprehension of how frequently little adjustments might alter the paths of the suggested dynamical system, ultimately opening the door to more precise and knowledgeable predictions of its behaviours under various circumstances.

### Sensitivity analysis

In this part, we focus on evaluating the sensitivity analysis of the governing system encapsulated by Eq. (43) in the context of numerical method namely, the Runge-Kutta method^36^. To do so, we simulate and study the dynamical model as follows:

82 $$\begin{aligned} \frac{d \Omega }{d\chi }= & {\mathcal {W}},\nonumber \\ \frac{dW}{d\chi }= & \mu _{1}\Omega (\chi )-\mu _{2}\Omega ^{3}(\chi ). \end{aligned}$$

**Fig. 7:**
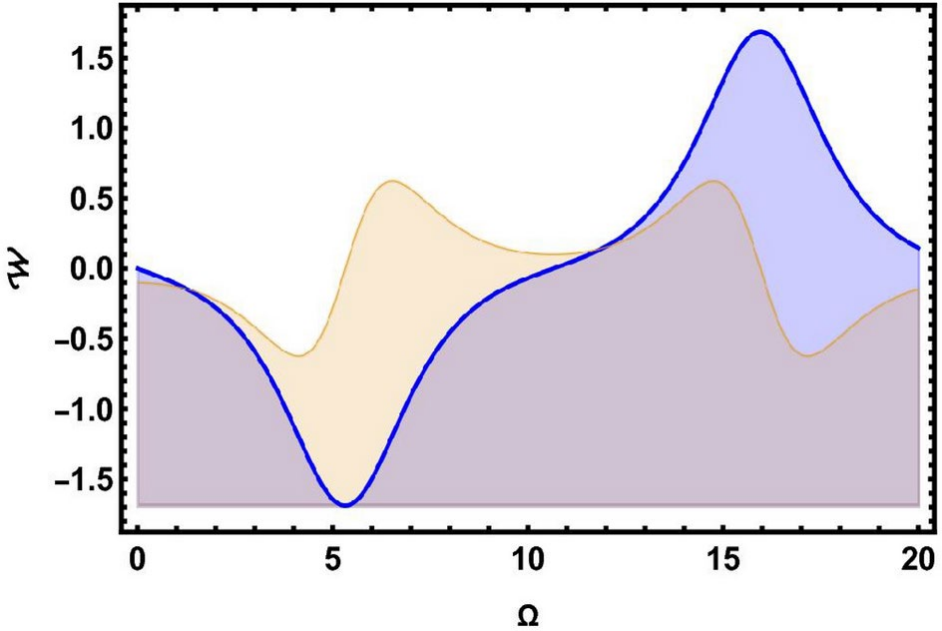
Sensitivity simulations portray of system (82) for initial conditions $(\Omega , {\mathcal {W}})=(0, 0)$ in blue curve and $(\Omega , {\mathcal {W}})=(0, -0.1)$ in yellow curve.

**Fig. 8:**
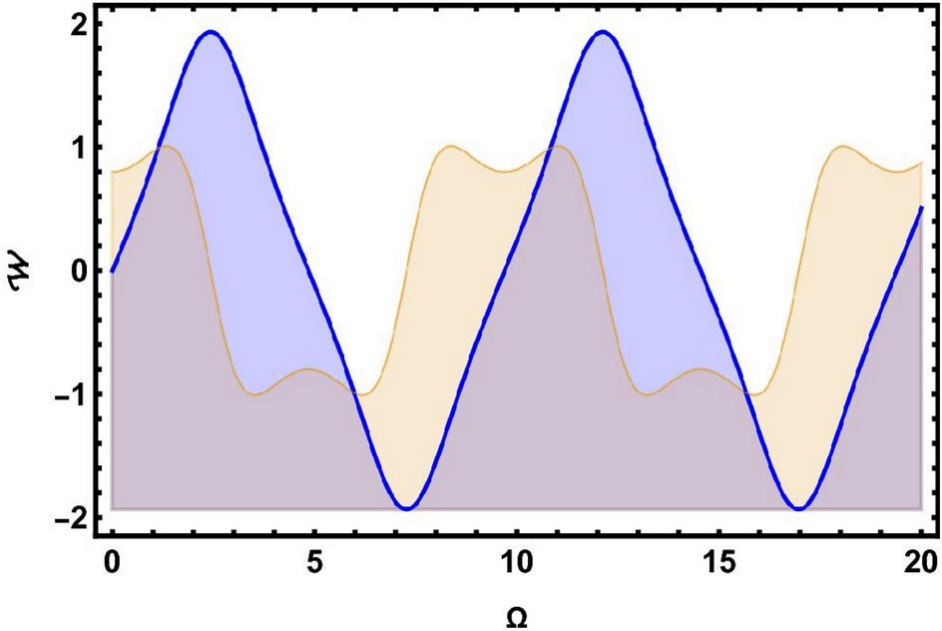
Sensitivity simulations portray of system (82) for initial conditions $(\Omega , {\mathcal {W}})=(0, 0)$ in blue curve and $(\Omega , {\mathcal {W}})=(0, 0.8)$ in yellow curve.

**Fig. 9:**
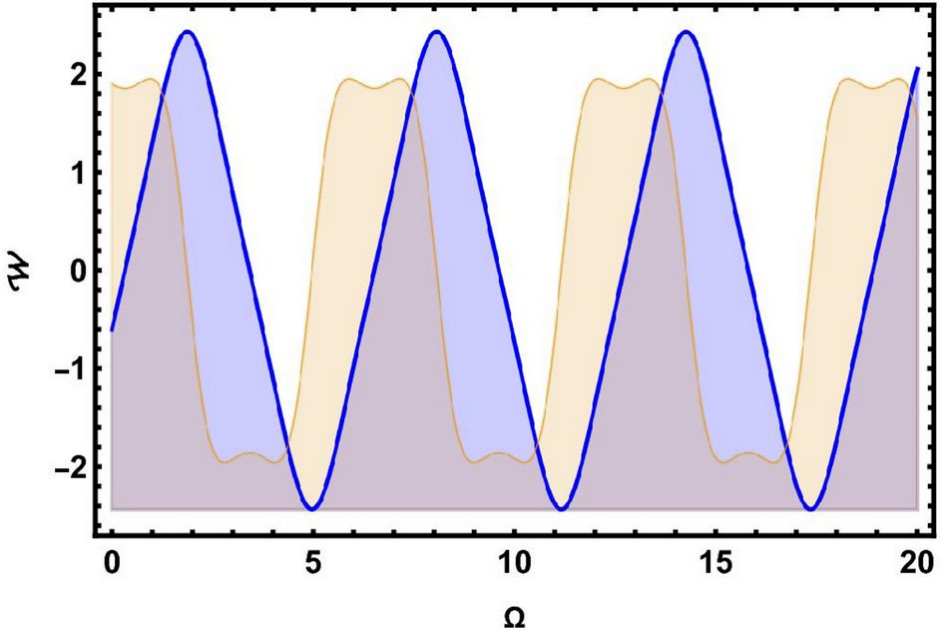
Sensitivity simulations portray of system (82) for initial conditions $(\Omega , {\mathcal {W}})=(-0.6, 0)$ in blue curve and $(\Omega , {\mathcal {W}})=(0, 1.9)$ in yellow curve.

**Fig. 10:**
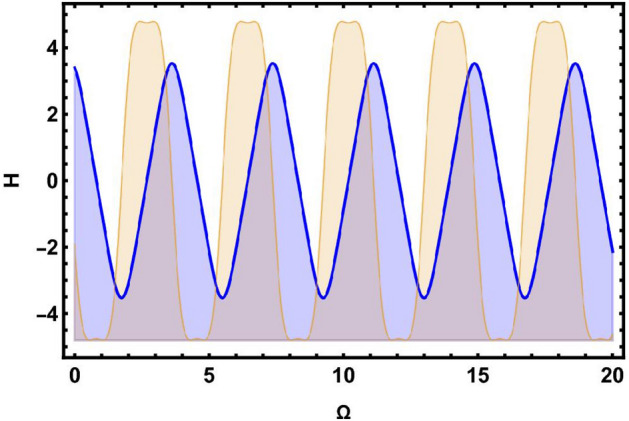
Sensitivity simulations portray of system (82) for initial conditions $(\Omega , {\mathcal {W}})=(3.4, 0)$ in blue curve and $(\Omega , {\mathcal {W}})=(0, 1.9)$ in yellow curve.

**Fig. 11:**
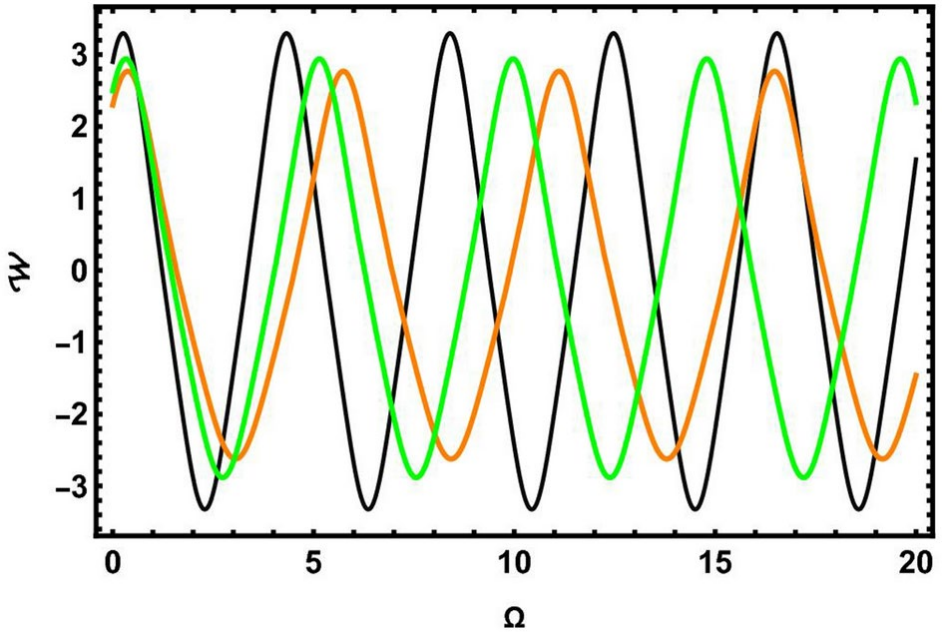
Sensitivity simulations portray of system (82) for initial conditions $(\Omega , {\mathcal {W}})=(2.9, 0)$ in black and $(\Omega , {\mathcal {W}})=(2.3, 0)$ in orange curve and $(\Omega , {\mathcal {W}})=(2.5, 0)$ in green curve.

Reviewing and contrasting the four solution curves using various parameter values, $\vartheta =0.2,~\wp _2=0.33,~\wp _3=0.4$, is illustrated in Figs. 7, 8, 9, 10 and 11. In Fig. 7, there are two solutions: $(\Omega , {\mathcal {W}})=(0, 0)$ in blue curve and $(\Omega , {\mathcal {W}})=(0, -0.1)$ in yellow curve. In Fig. 8, there are two solutions: $(\Omega , {\mathcal {W}})=(0, 0)$ in blue curve and $(\Omega , {\mathcal {W}})=(0, 0.8)$ in yellow curve. Fig. 9 demonstrates two solutions: $(\Omega , {\mathcal {W}})=(-0.6, 0)$ in blue curve and $(\Omega , {\mathcal {W}})=(0, 1.9)$ in yellow curve. Similarly, Fig. 10 elaborates two solutions: $(\Omega , {\mathcal {W}})=(3.4, 0)$ in blue curve and $(\Omega , {\mathcal {W}})=(0, 1.9)$ in yellow curve. Similarly, Fig. 11 signifies three solutions: $(\Omega , {\mathcal {W}})=(2.9, 0)$ in black and $(\Omega , {\mathcal {W}})=(2.3, 0)$ in orange curve and $(\Omega , {\mathcal {W}})=(2.5, 0)$ in green curve. Sensitivity analysis is essential for enhancing the reliability, robustness, and applicability of models across various domains. By systematically assessing the sensitivity of model outcomes to input parameters, researchers and decision-makers can make informed decisions, manage risks, and improve the overall quality of their analyses and predictions.

## Simulation and discussion

**Fig. 12:**
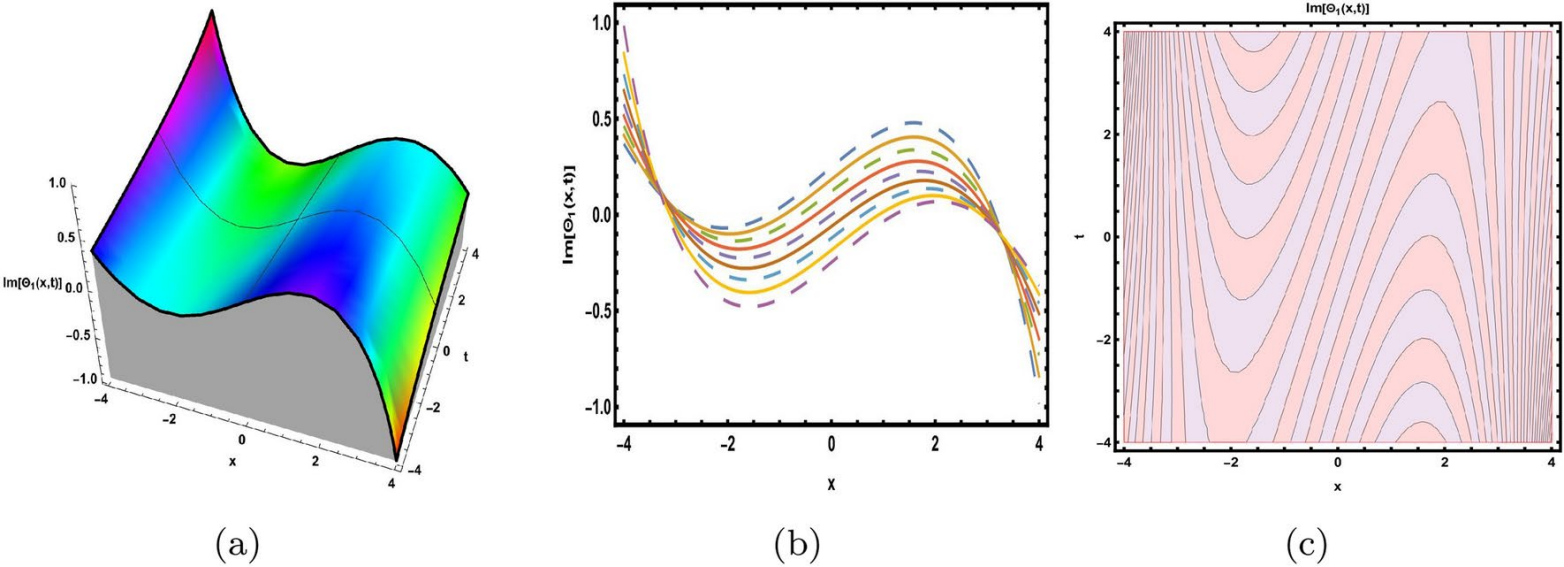
Graphical visualization of solution (46) with arbitrary parameters $\gamma _1=0.7,~\gamma _2=-0.2,~\gamma _3=0.1$, $\wp _2=0.8,~\wp _3=0.9,~\delta =0.07,~\alpha =1,~\beta =0.1,~a=0.1,~\kappa =-0.55$.

**Fig. 13:**
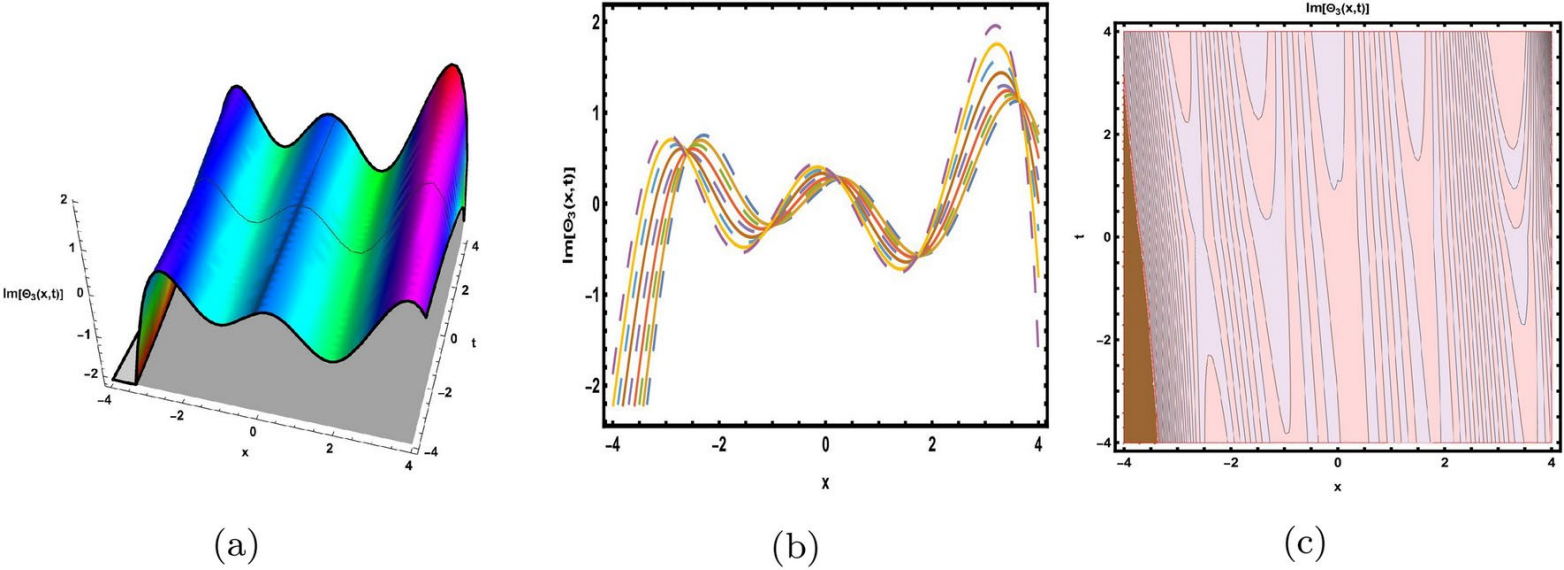
Graphical visualization of solution (48) with arbitrary parameters $\gamma _1=0.6,~\gamma _2=0.61,~\gamma _3=0.28$, $\wp _3=0.7,~\delta =0.57,~\alpha =0.9,~\beta =0.1,~a=0.2,~\kappa =2.1,~m=0.5,~n=0.8$.

**Fig. 14:**
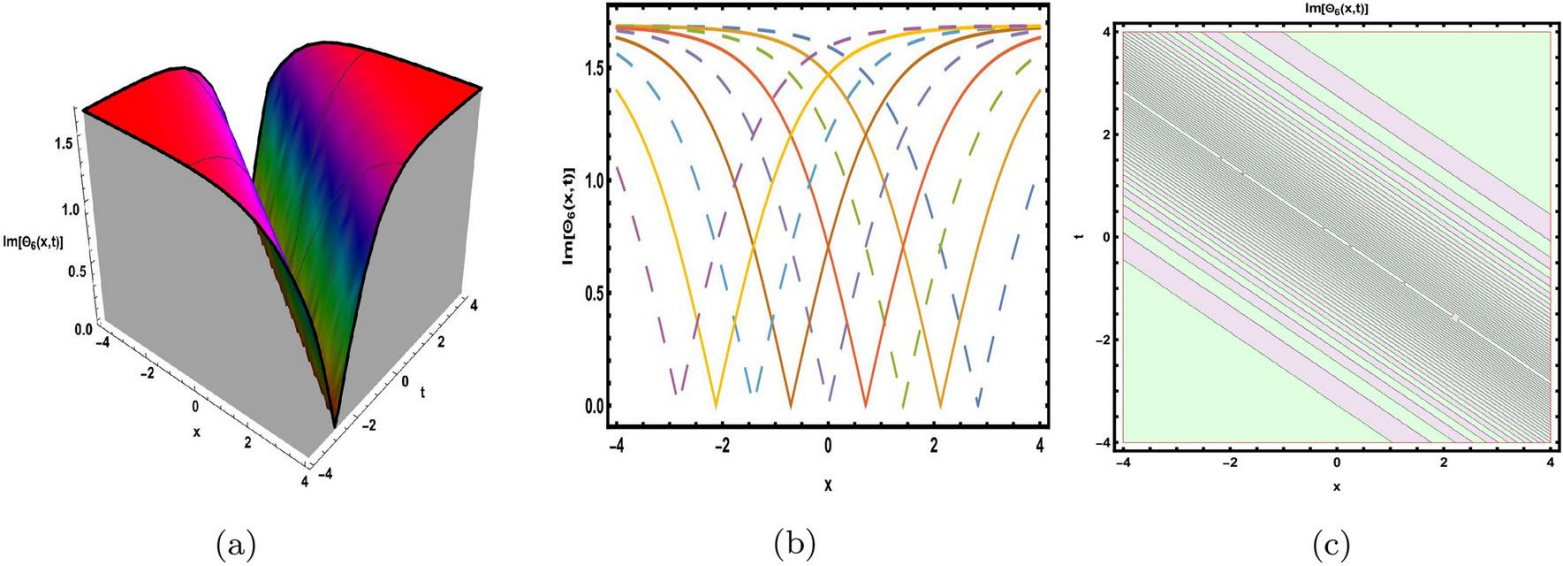
Graphical visualization of solution (51) with arbitrary parameters $\gamma _1=0.4,~\gamma _2=0.6,~\gamma _3=-0.4$, $\wp _2=0.3,~\wp _3=0.42,~\delta =-0.5,~\alpha =1,~\beta =0.6,~a=0.23,~\kappa =-0.9$.

**Fig. 15:**
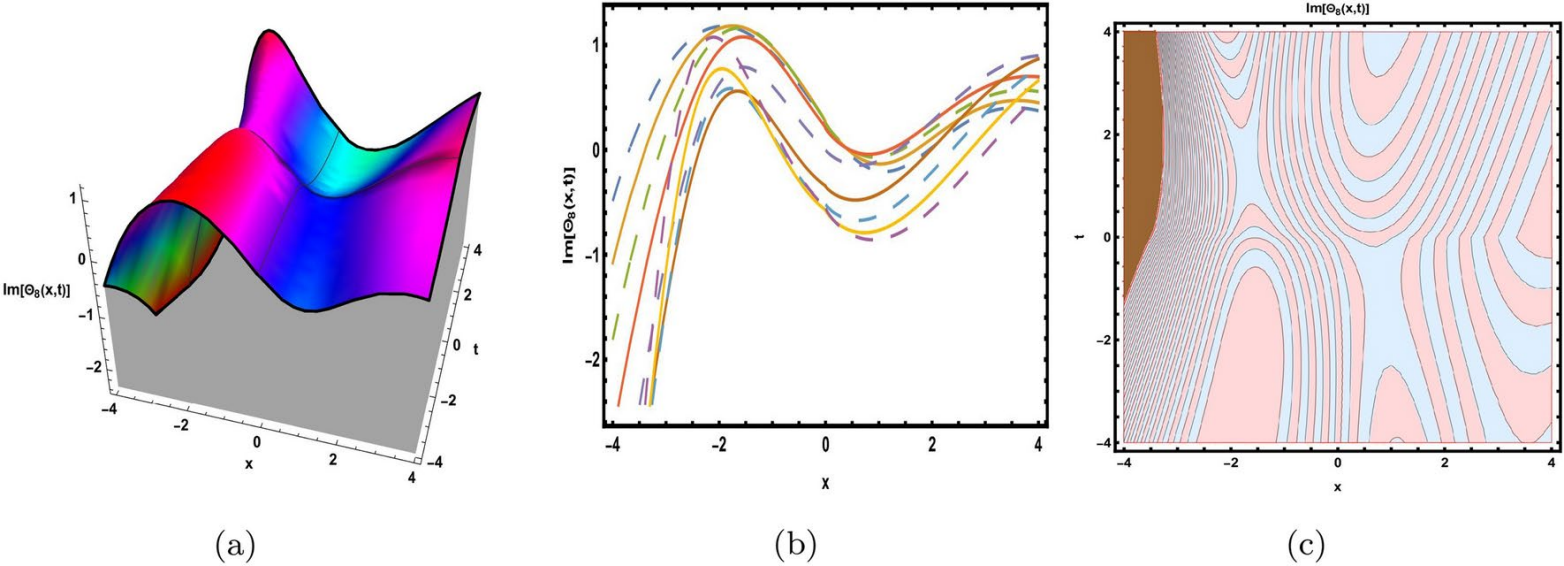
Graphical visualization of solution (53) with arbitrary parameters $\gamma _1=0.22,~\gamma _2=0.24,~\gamma _3=-0.26$, $~\wp _2=0.3,~\wp _3=0.2, delta =-0.5,~\alpha =0.8,~\beta =0.6,~a=0.3,~\kappa =0.9,~m=0.2,~n=0.3$.

**Fig. 16:**
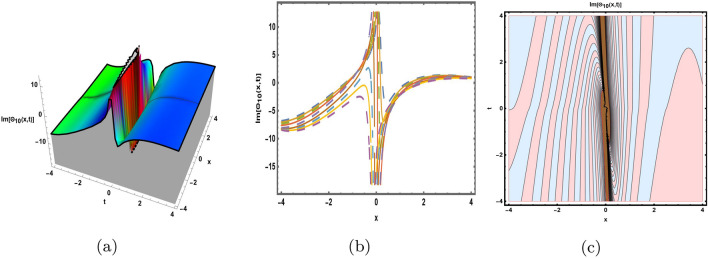
Graphical visualization of solution (55) with arbitrary parameters $\gamma _1=0.2,~\gamma _2=0.4,~\gamma _3=0.6$, $\wp _2=0.83,~\wp _3=0.32,~\delta =-0.5,~\alpha =0.8,~\beta =0.6,~a=0.3,~\kappa =0.9,~m=0.2,~n=0.3$.

**Fig. 17:**
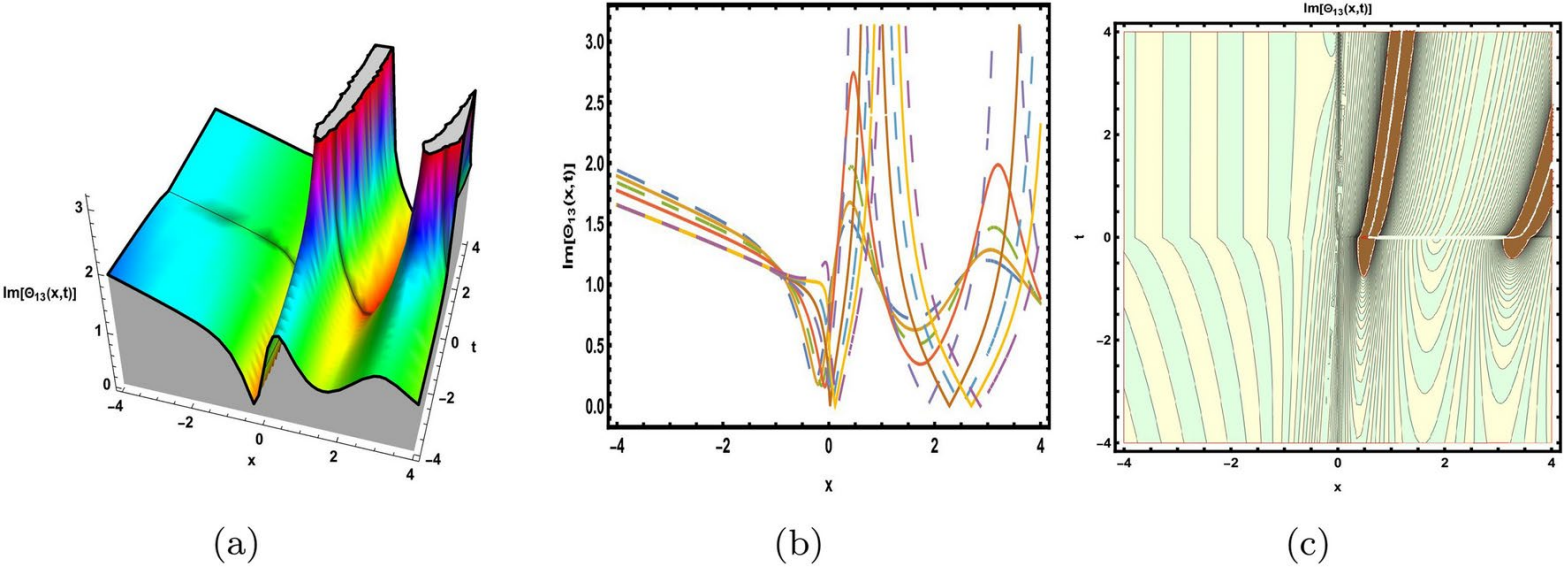
Graphical visualization of solution (58) with arbitrary parameters $\gamma _1=-0.9,~\gamma _2=0,~\gamma _3=-0.6$, $\wp _2=0.6,~\wp _3=0.8, \delta =-0.26,~\alpha =0.6,~\beta =0.8,~a=0.5,~\kappa =0.2,~m=0.1,~n=0.3$.

**Fig. 18:**
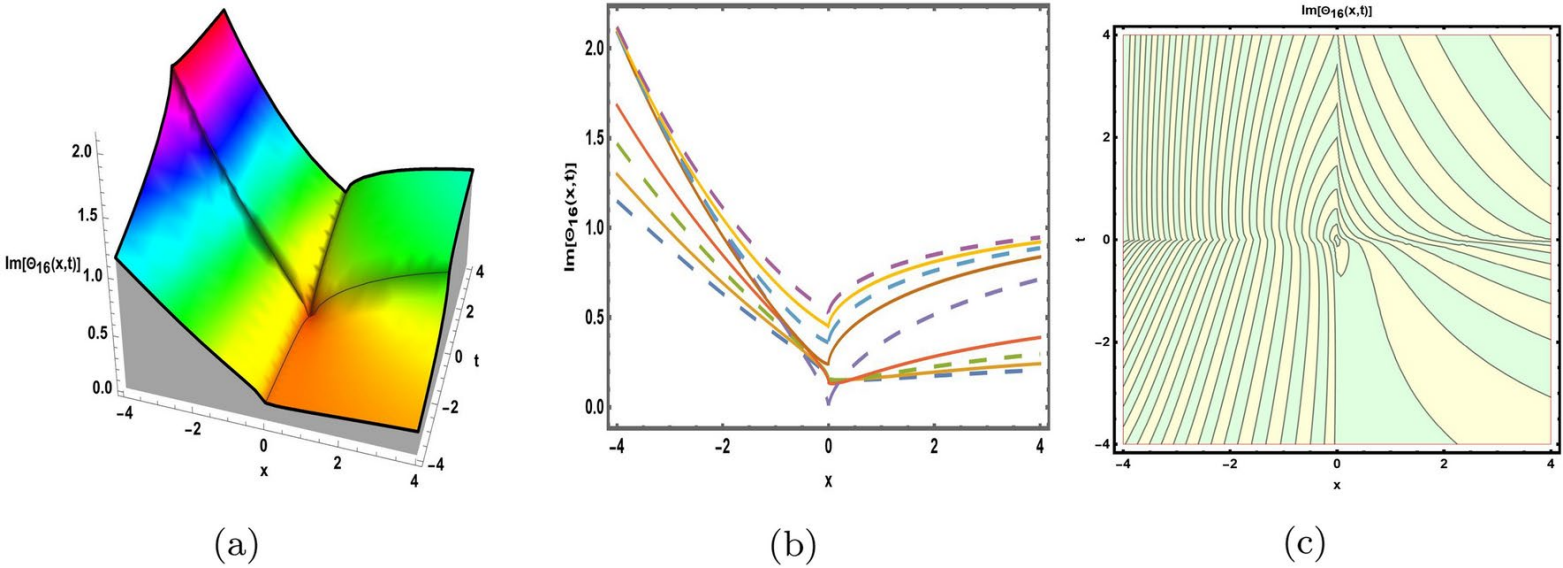
Graphical visualization of solution (61) with arbitrary parameters $\gamma _1=-0.29,~\gamma _2=0,~\gamma _3=0.16$, $\wp _2=0.26,~\wp _3=0.28,~\delta =-0.6,~\alpha =0.6,~\beta =0.8,~a=0.15,~\kappa =0.22$.

**Fig. 19:**
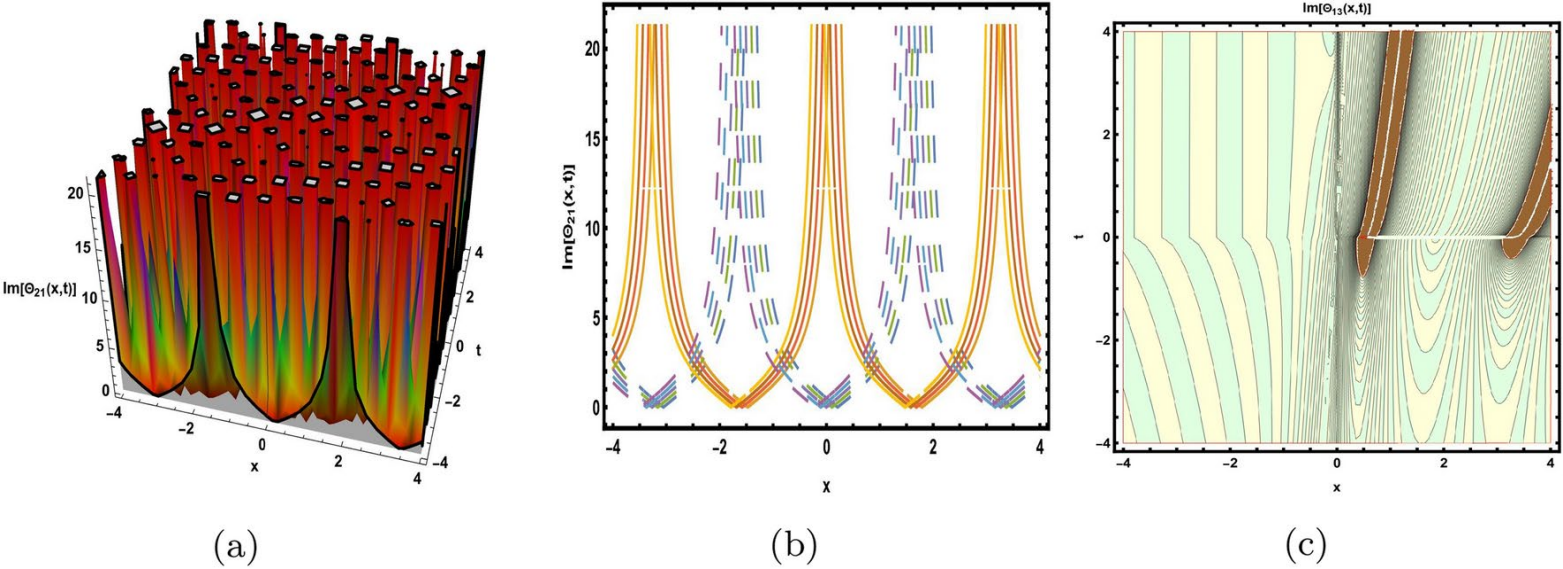
Graphical visualization of solution (66) with arbitrary parameters $\gamma _1=1,~\gamma _2=0,~\gamma _3=1$, $\wp _2=0.69,~\wp _3=0.9,~\delta =0.54,~\alpha =1,~\beta =0.9,~a=0.2,~\kappa =-0.9$.

**Fig. 20:**
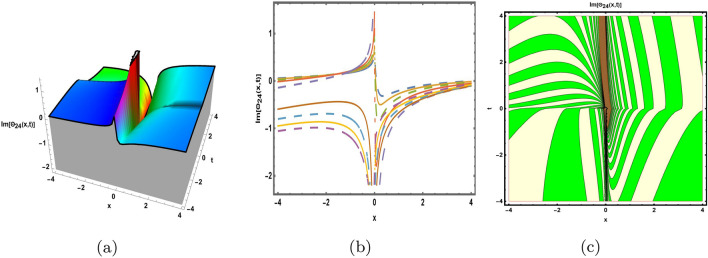
Graphical visualization of solution (69) with arbitrary parameters $\gamma _1=0.16,~\gamma _2=0,~\gamma _3=0.16$, $\wp _2=0.46,~\wp _3=0.8, \delta =-1.6,~\alpha =0.6,~\beta =0.8,~a=0.15,~\kappa =0.22,~m=0.1,~n=0.2$.

**Fig. 21:**
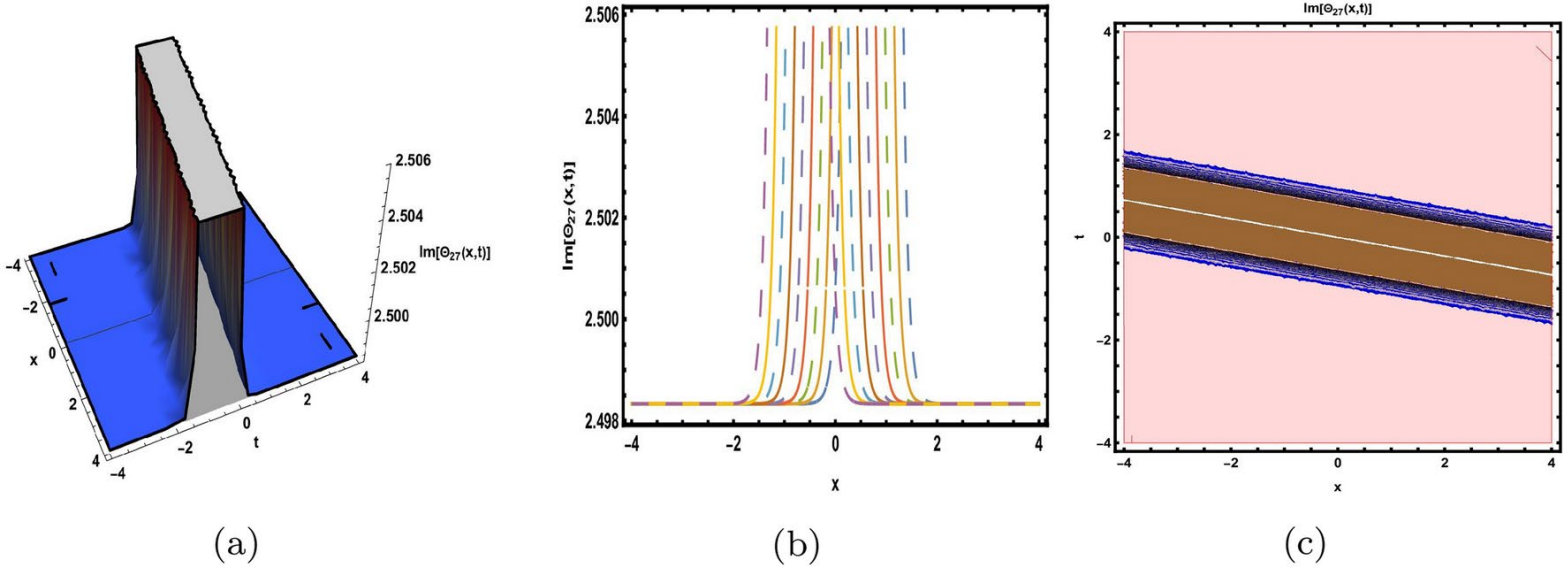
Graphical visualization of solution (72) with arbitrary parameters $\gamma _1=1,~\gamma _2=0,~\gamma _3=-1$, $\wp _2=0.69,~\wp _3=0.9, \delta =0.54,~\alpha =1,~\beta =0.9,~a=0.2,~\kappa =-0.9$.

**Fig. 22:**
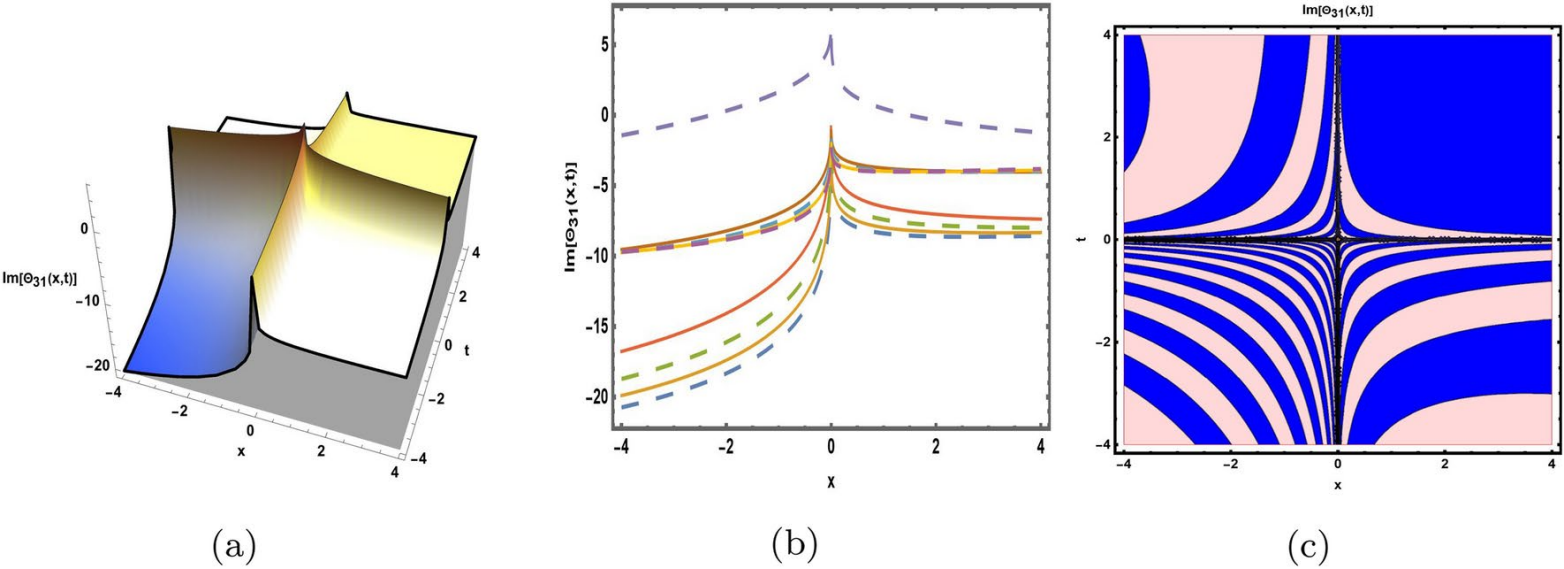
Graphical visualization of solution (76) with arbitrary parameters $\gamma _1=1,~\gamma _2=2,~\gamma _3=1$, $\wp _2=0.3,~\wp _3=0.6,~\delta =0.76,~\alpha =0.16,~\beta =0.8,~a=0.15,~\kappa =0.22$.

This part presents a detailed comparison between the evaluated finding and the previously calculated results, elaborating on the unique contribution to the research. In recent research, analytical methods for solving the third order FNLSE have been given. To be more specific, Günhan Ay and Yaşar^29^ have esed the extended modified auxiliary equation mapping method to get a variety of precise single wave solutions. However, our work has painstakingly derived several new accurate solitary wave solutions for the third-order FNLSE, a crucial mathematical model in light propagation in optical fibers during the generation of ultrashort pulses, by encircling the NEDAM. New insights into the properties of nonlinear dispersive waves that are prone to high-order effects are provided by these analyzed soliton solutions. It is evident from the literature that a variety of soliton structures cannot be created without the selected technique. Furthermore, we employ the Hamiltonian property, which measures the accuracy of the solutions, to methodically examine the stability requirements of every single one of our found solitary wave solutions. Also, the bifurcation analysis and sensitive analysis of the above mentioned model is also investigated. A crucial tool for assessing the physical features of the model, explaining nonlinear behaviour, and verifying the accuracy of analytical approaches is the graphical representation of solitary wave solutions. As demonstrated in Figs. 12, 13, 14, 15, 16, 17, 18, 19, 20, 21 and 22, the dynamical perspective of graphical behavior for periodic, dark, singular, and composite optical soliton solutions provides new insights into the properties of nonlinear dispersive waves under the influence of high-order effects. Figure 12 presents the trigonometric solution of Eq. (46) obtained via the NEDAM method, visualized through 3D, 2D, and contour plots. This solution represents oscillatory behavior, which is characteristic of systems where wave interactions lead to periodic variations in amplitude and phase, often observed in waveguides and resonant structures. Figure 13 showcases mixed trigonometric solutions of Eq. (48), also using 3D, 2D, and contour formats. These solutions illustrate complex oscillations resulting from the interplay of multiple trigonometric functions, which can model phenomena like modulated waves in nonlinear media. Figure 14 depicts the dark soliton solutions of Eq. (51). These solutions are characterized by localized drops in intensity within a continuous wave background, which is typical in optical fibers and fluid dynamics where they represent areas of destructive interference. Figure 15 illustrates the bright-dark soliton solutions of Eq. (53), which represent interactions between bright and dark solitons. These solutions are crucial for understanding energy transfer in nonlinear optical systems, where bright solitons represent regions of high intensity and dark solitons represent low-intensity troughs. Figure 16 signifies the mixed hyperbolic solutions of Eq. (55), derived using appropriate parameters. These solutions reflect the hyperbolic waveforms that occur in scenarios like shock waves and solitary waves in plasmas, where the waveform maintains its shape over time. Figure 17 demonstrates the periodic-singular behavior of solitons in Eq. (58). This behavior is indicative of solutions that oscillate periodically with singularities, which can occur in highly nonlinear systems where energy concentration leads to extreme events. Figure 18 denotes the dark solution of Eq. (61), emphasizing regions of reduced amplitude within a wave train. This is particularly relevant in nonlinear optics, where such solutions can describe stable, localized intensity dips in a propagating wave. Figure 19 illustrates the periodic solution of Eq. (66), which represents regular, repeating patterns in waveforms, commonly seen in systems with inherent periodicity such as crystals or resonant cavities. Figure 20 depicts the combo soliton solutions of Eq. (61), showcasing the interaction of multiple solitons. These solutions highlight the complex dynamics of soliton interactions, which are essential for understanding the stability and evolution of soliton trains in various physical contexts. Figure 21 represents the singular behavior of Eq. (72), where the solutions exhibit singularities, indicating points of infinite amplitude. This behavior is typical in systems that experience abrupt changes, such as in the formation of shock waves or rogue waves. Figure 22 illustrates the rational solution of Eq. (76), which is characterized by polynomial expressions in space and time. These solutions are often associated with rogue waves or other localized phenomena in nonlinear dispersive media, where they describe rare, high-amplitude events. Scholars can gain insights on the behavior of individual waves by the visualization of these waves for a variety of purposes. This research clarifies nonlinear processes in dispersive waves, which are relevant to optical fibres and practical physics.

## Conclusion

In this study, we have rigorously explored the behavior of the third-order FNLSE using a novel approach, the NEDAM. We successfully identified a diverse array of exact wave structures for the selected model, including dark, combo, singular, hyperbolic, planar, singular periodic waves, and mixed trigonometric forms. Also, by integrating sensitivity and bifurcation analyses into our study, we can gain deeper insights into the behavior of the third-order FNLSE and further validate the effectiveness of the NEDAM. Our research offers significant insights into the soliton solutions and nonlinear dynamics associated with the FNLSE when combined with the *M*-truncated derivative. These insights enhance our understanding of complex nonlinear wave processes. Our findings improve upon previous work in several critical ways. First, by employing NEDAM, we have broadened the scope of exact solutions that can be obtained, uncovering new wave structures that were not accessible through earlier methods. This represents a substantial advancement in the analytical capabilities available for studying the FNLSE. Second, our rigorous investigation into the stability and resilience of these solutions confirms their practical reliability over specific intervals, a crucial aspect that bolsters the potential for real-world applications. The practical implications of our work extend across multiple fields, including nonlinear optics and fluid dynamics, underscoring the versatility and significance of the third-order FNLSE. Additionally, this study lays the groundwork for future research, where we can explore the exact soliton solutions of the FNLSE under the influence of multiplicative or additive color noise.

## Data Availability

The datasets used and/or analysed during the current study available from the corresponding author on reasonable request.
